# Supplementary material for: Longitudinal population-level HIV epidemiologic and genomic surveillance highlights growing gender disparity of HIV transmission in Uganda
Source: Nat Microbiol. 2023 Dec 5;9(1):35–54. doi: 10.1038/s41564-023-01530-8 (PMC10769880; doi:10.1038/s41564-023-01530-8)
Supplement: Supplementary file 1 — Supplementary Figs. 1–5 and Tables 1–10. [file 41564_2023_1530_MOESM1_ESM.pdf]

nature microbiology

Article

https://doi.org/10.1038/s41564-023-01530-8

Longitudinal population-level HIV epidemiologic and genomic surveillance highlights growing gender disparity of HIV transmission in Uganda

In the format provided by the authors and unedited

List of Figures

S1 **Flowchart of census eligible individuals through to individuals in the incidence and transmission cohorts.** S2

S2 **Schema illustrating the refinement of phylogenetic time since infection estimates.** S6

S3 **Comparison of incidence rate estimates under an individual-level additive effects Poisson regression model and a population-level LOESS model with independent age effects in each survey round.** (**a**) Mean and 95% uncertainty ranges of longitudinal age-specific incidence rates obtained with the individual-level additive effects Poisson regression model used in the central analysis (**b**) Same using a population-level LOESS model with independent age effects in each survey round. S7

S4 **Comparison of incidence rate estimated on data containing all communities and data subset to 28 continuously surveyed communities** (**a**) Mean and 95% uncertainty ranges of longitudinal age-specific incidence rates estimated on data from all communities surveyed (**b**) Same using data subset to 28 continuously surveyed communities. S8

S5 **Sensitivity in estimating the age of transmitting partners to right censoring of likely transmission pairs.** Posterior median (line) and 95% credible interval (ribbon) of the age of male transmitting partners by the age of the infected female (x-axis) by survey round (row facet) for the central and sensitivity analyses (column facet). Median and 95% credible interval of the age of male transmitting partners across the age of the infected female is indicated with a triangle and an error bar. S9

List of Tables

S1 **Characteristics of the RCCS study population.** S11

S2 **Communities surveyed by RCCS in rounds 10-18.** S13

S3 **Characteristics of the longitudinal HIV incidence cohort.** S14

S4 **Model comparison for estimating longitudinal, age-specific incidence rates.** S16

S5 **Longitudinal HIV deep-sequencing.** S17

S6 **Model comparison for estimating longitudinal, age-specific transmission flows.** S19

S7 **Longitudinal HIV transmission flows by age and gender.** S20

S8 **Sexual behaviour characteristics in RCCS participants, round 15, October 08 2011 - July 05 2013.** S21

S9 **Self-reported ART use and viral suppression in RCCS participants with HIV.** S22

S10 **Sensitivity analyses.** S23

Rakai Health Sciences Program consortium members (all)

Larry W Chang[3,2,11], Ronald M Galiwango[2], M Kate Grabowski[2,14,11], Ronald H Gray[13], Jade C Jackson[14], Joseph Kagaayi[2], Edward Nelson Kankaka[3,4], Godfrey Kigozi[2], Oliver Laeyendecker[15,16], Thomas C Quinn[14,15,16], Steven J. Reynolds[2,15,16], John Santelli[18], David Serwadda[2,19], Nelson K. Sewankambo[19], Joseph Ssekasanvu[11], Robert Ssekubugu[2], Victor Ssempijja[5,6], Maria J Wawer[2,11], Dorean Nabukalu[2], Anthony Ndyanabo[2], Joseph Ssekasanvu[2], Hadijja Nakawooya[2], Jessica Nakukumba[2], Grace N. Kigozi[2], Betty S. Nantume[2], Nampijja Resty[2], Jedidah Kambasu[2], Margaret Nalugemwa[2], Regina Nakabuye[2], Lawrence Ssebanobe[2], Justine Nankinga[2], Adrian Kayiira[2], Gorreth Nanfuka[2], Ruth Ahimbisibwe[2], Stephen Tomusange[2], Sarah Kalibbali[2], Margaret Nakalanzi[2], Joseph Ouma Otobi[2], Denis Ankunda[2], Joseph Lister Ssembatya[2], John Baptist Ssemanda[2], Robert Kairania[2], Emmanuel Kato[2], Alice Kisakye[2], James Batte[2], James Ludigo[2], Abisagi Nampijja[2], Steven Watya[2], Kighoma Nehemia[2], Sr. Margaret Anyokot[2], Joshua Mwinike[2], George Kibumba[2], Paschal Ssebowa[2], George Mondo[2], Francis Wasswa[2], Agnes Nantongo[2], Rebecca Kakembo[2], Josephine Galiwango[2], Geoffrey Ssemango[2], Andrew D. Redd[2], Caitlin E. Kennedy[2], Jennifer Wagman[2], Philip Kreniske[2], Nelson Sewankambo[2]

PANGEA-HIV-2 consortium members (all)

Lucie Abeler Dörner[7], Helen Ayles[29,23], David Bonsall[9,10], Rory Bowden[9,24], Vincent Calvez[25], Max Essex[26], Sarah Fidler[22,27], Christophe Fraser[10,7], Tanya Golubchik[12,7], M Kate Grabowski[2,14,11], Ravindra Gupta[28], Richard Hayes[22,20], Joshua Herbeck[30], Joseph Kagaayi[2], Pontiano Kaleebu[31], Jairam Lingappa[30], Sikhulile Moyo[26], Vladimir Novitsky[32], Thumbi Ndung'u[33], Deenan Pillay[34], Thomas C Quinn[14,15,16], Andrew Rambaut[35], Oliver Ratmann[1], Janet Seeley[31,36], Deogratius Ssemwanga[31] & Frank Tanser[33], Maria J Wawer[2,11]

[23] Zambart, University of Zambia, Ridgeway Campus, Lusaka, Zambia.
[24] Department of Statistics, Oxford University, Oxford, United Kingdom.
[25] Institut Pasteur, Paris, France.
[26] Botswana Harvard AIDS Institute Partnership, Gaborone, Botswana.
[27] Department of Infectious Disease, Imperial College London, London, United Kingdom.
[28] Department of Medicine, University of Cambridge, Cambridge, United Kingdom.
[29] Department of Infectious Disease Epidemiology and International Health, The London School of Hygiene & Tropical Medicine, London, United Kingdom.
[30] University of Washington, Seattle, Washington, United States.
[31] MRC/UVRI, Entebbe, Uganda.
[32] Harvard University, Cambridge, Massachusetts, United States.
[33] Africa Health Research Institute (AHRI), Durban, South Africa.

[34] University College London, London, United Kingdom.
[35] University of Edinburgh, Edinburgh, United Kingdom.
[36] Department of Global Health and Development, The London School of Hygiene & Tropical Medicine, London, United Kingdom.

S1 Supplementary Figures

S2 LIST OF TABLES

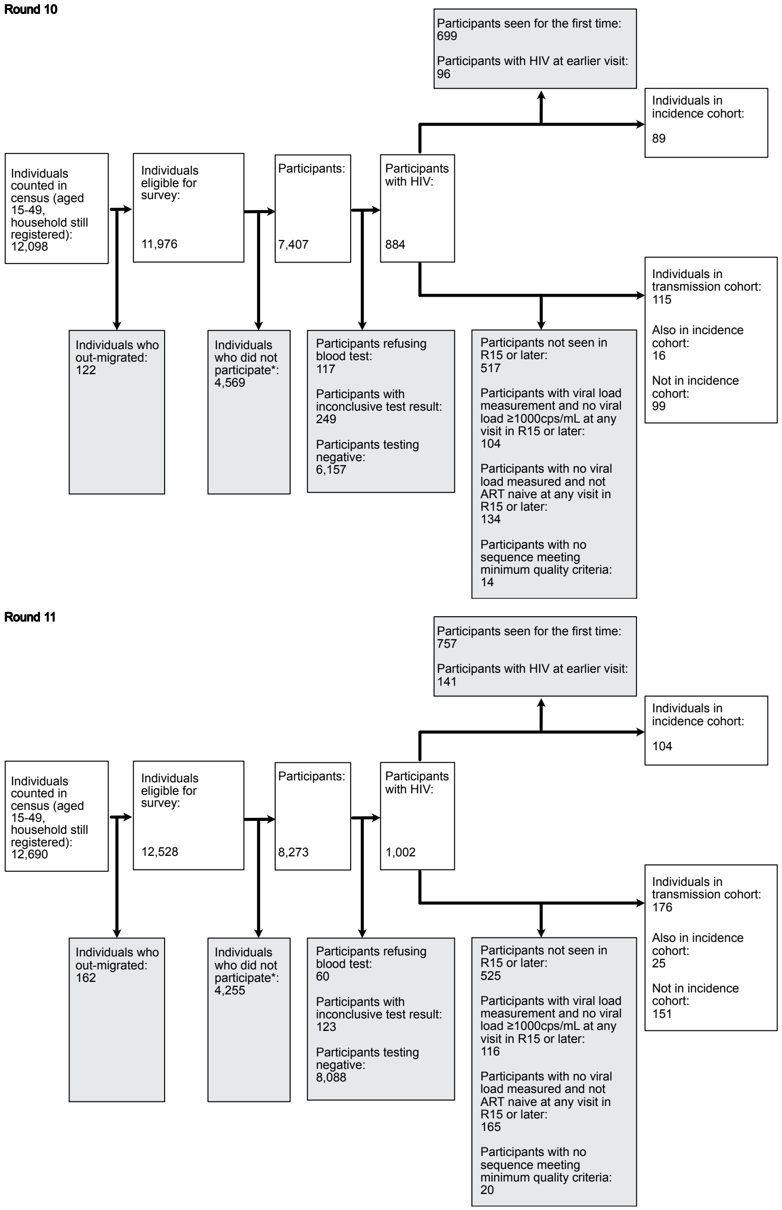

Supplementary Fig. S1: **Flowchart of census eligible individuals through to individuals in the incidence and transmission cohorts.**

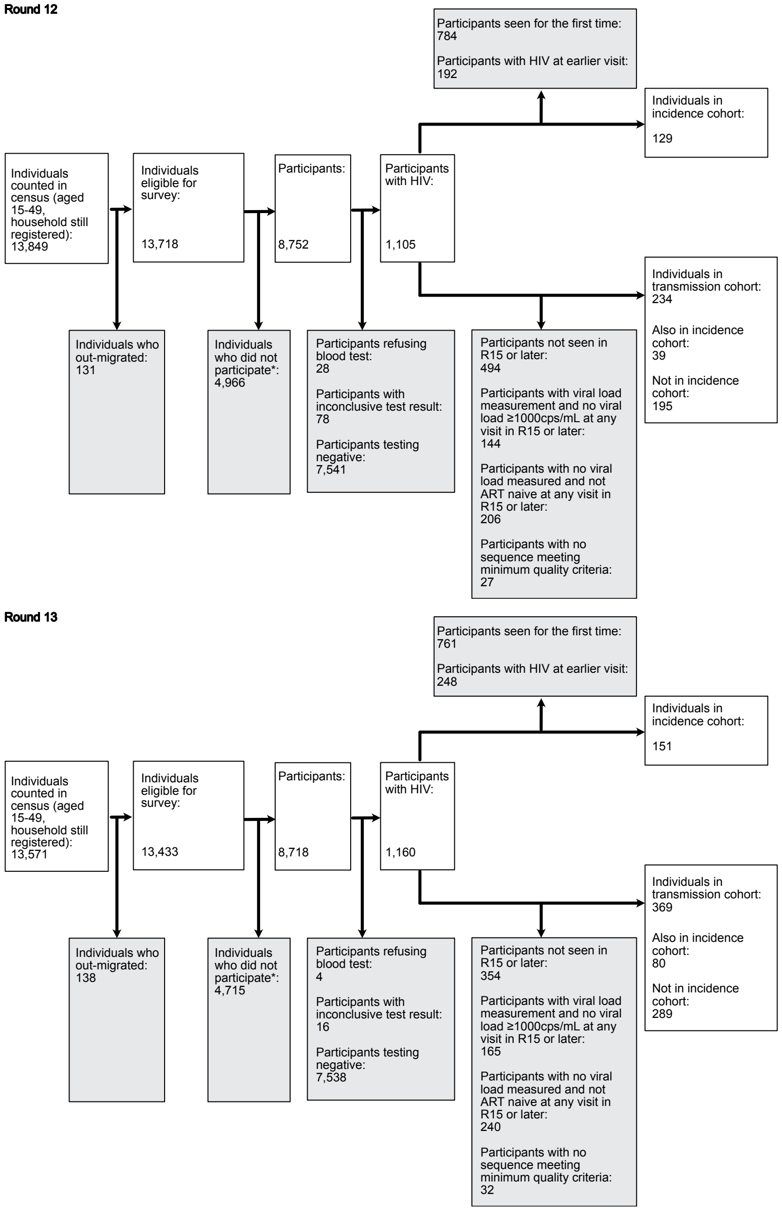

Supplementary Fig. S1: **Flowchart of census eligible individuals through to individuals in the incidence and transmission cohorts.** (cont.)

S4 LIST OF TABLES

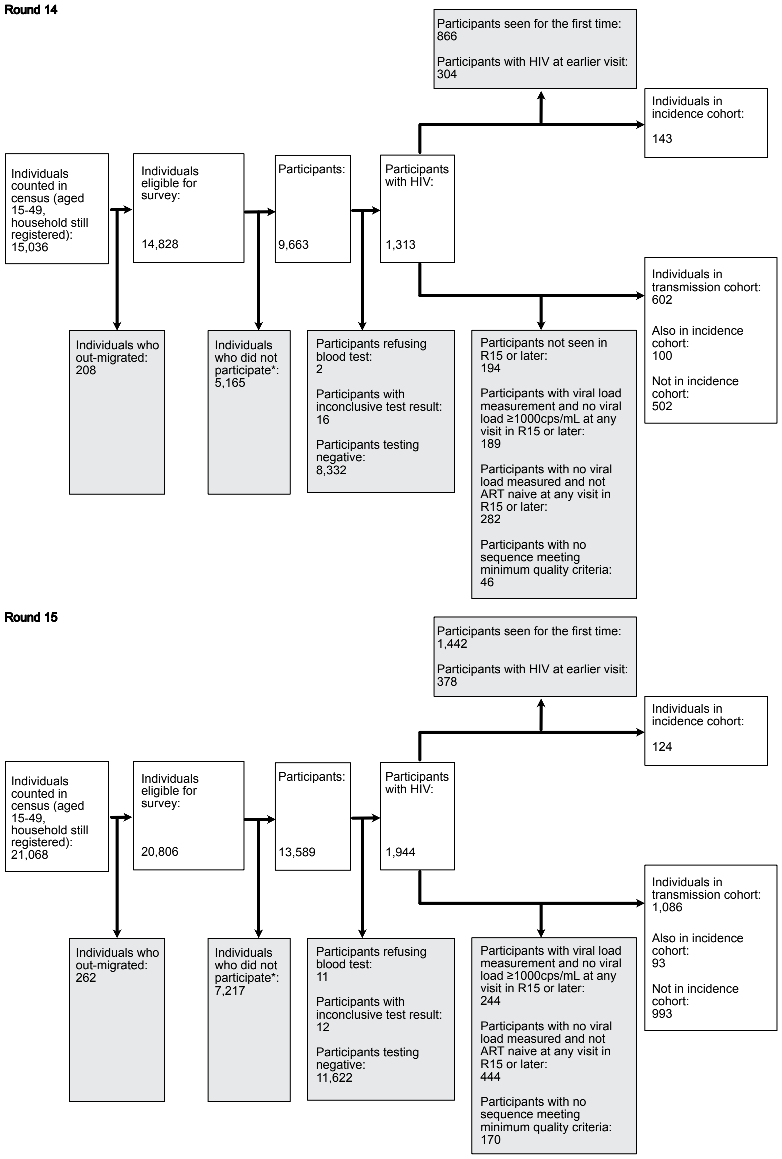

Supplementary Fig. S1: **Flowchart of census eligible individuals through to individuals in the incidence and transmission cohorts.** (cont.)

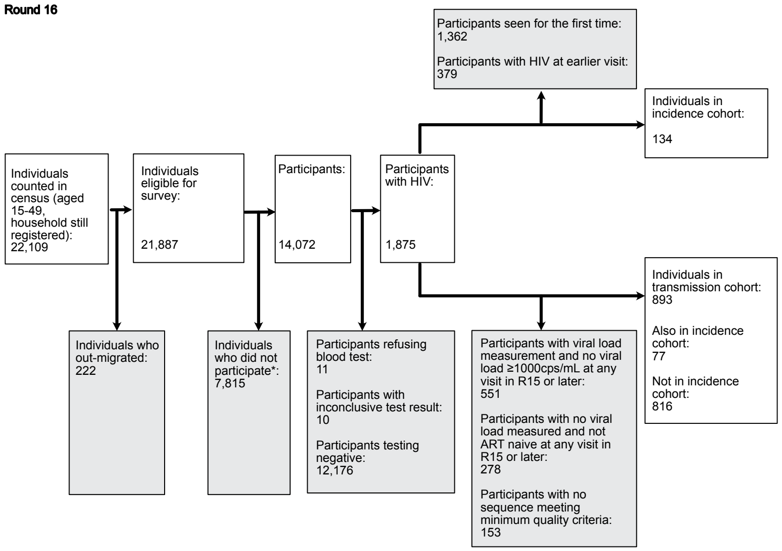

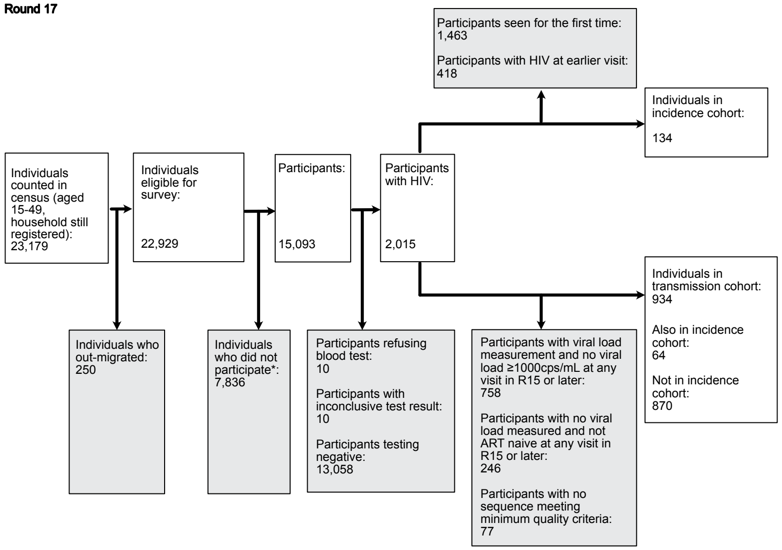

Supplementary Fig. S1: **Flowchart of census eligible individuals through to individuals in the incidence and transmission cohorts.** (cont.)

S6 LIST OF TABLES

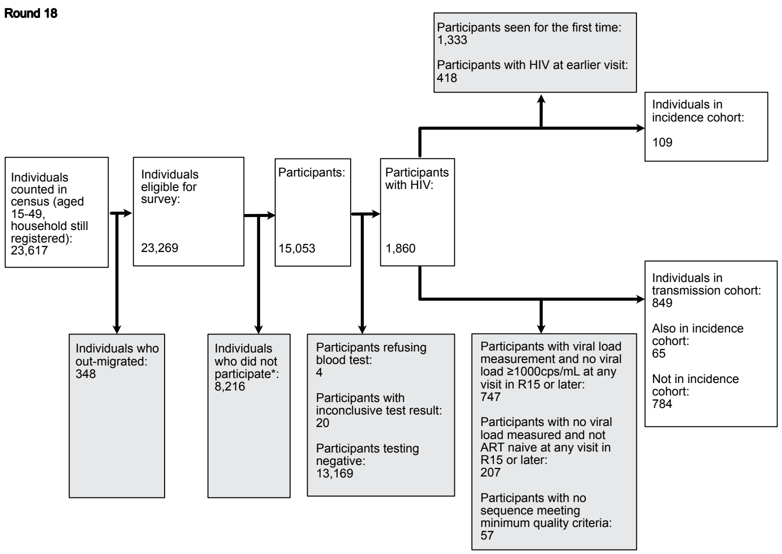

Supplementary Fig. S1: Flowchart of census eligible individuals through to individuals in the incidence and transmission cohorts. (cont.)

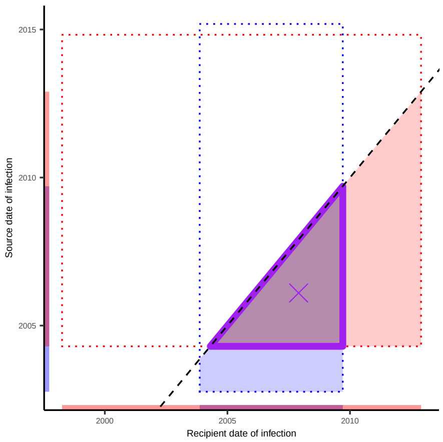

Supplementary Fig. S2: Schema illustrating the refinement of phylogenetic time since infection estimates.

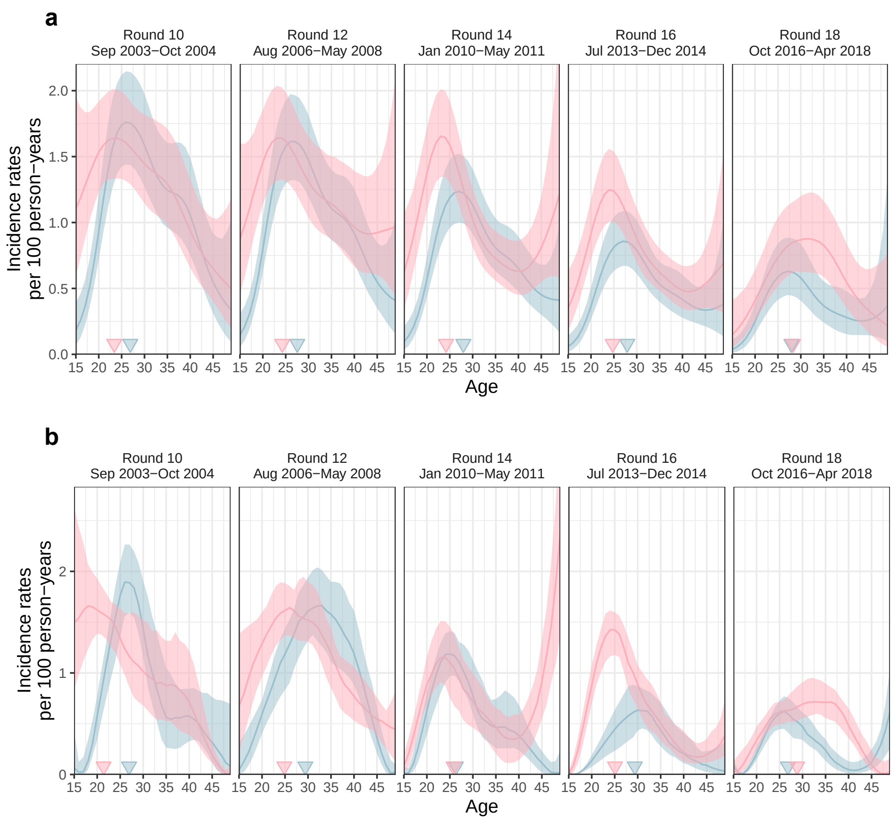

Supplementary Fig. S3: **Comparison of incidence rate estimates under an individual-level additive effects Poisson regression model and a population-level LOESS model with independent age effects in each survey round.** (**a**) Mean and 95% uncertainty ranges of longitudinal age-specific incidence rates obtained with the individual-level additive effects Poisson regression model used in the central analysis (**b**) Same using a population-level LOESS model with independent age effects in each survey round.

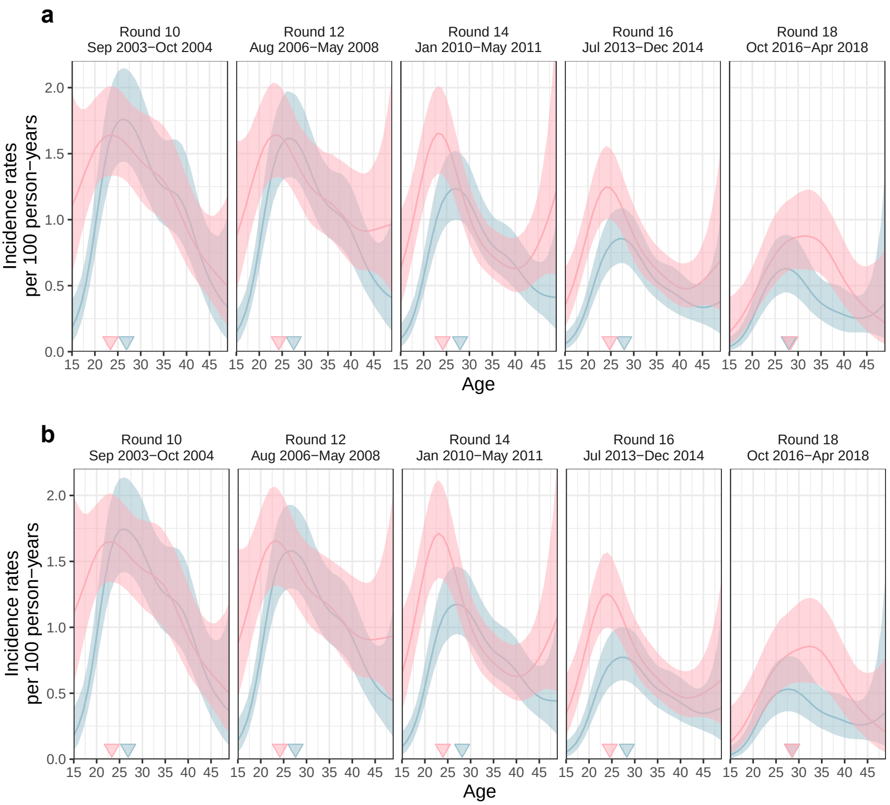

Supplementary Fig. S4: **Comparison of incidence rate estimated on data containing all communities and data subset to 28 continuously surveyed communities** (**a**) Mean and 95% uncertainty ranges of longitudinal age-specific incidence rates estimated on data from all communities surveyed (**b**) Same using data subset to 28 continuously surveyed communities.

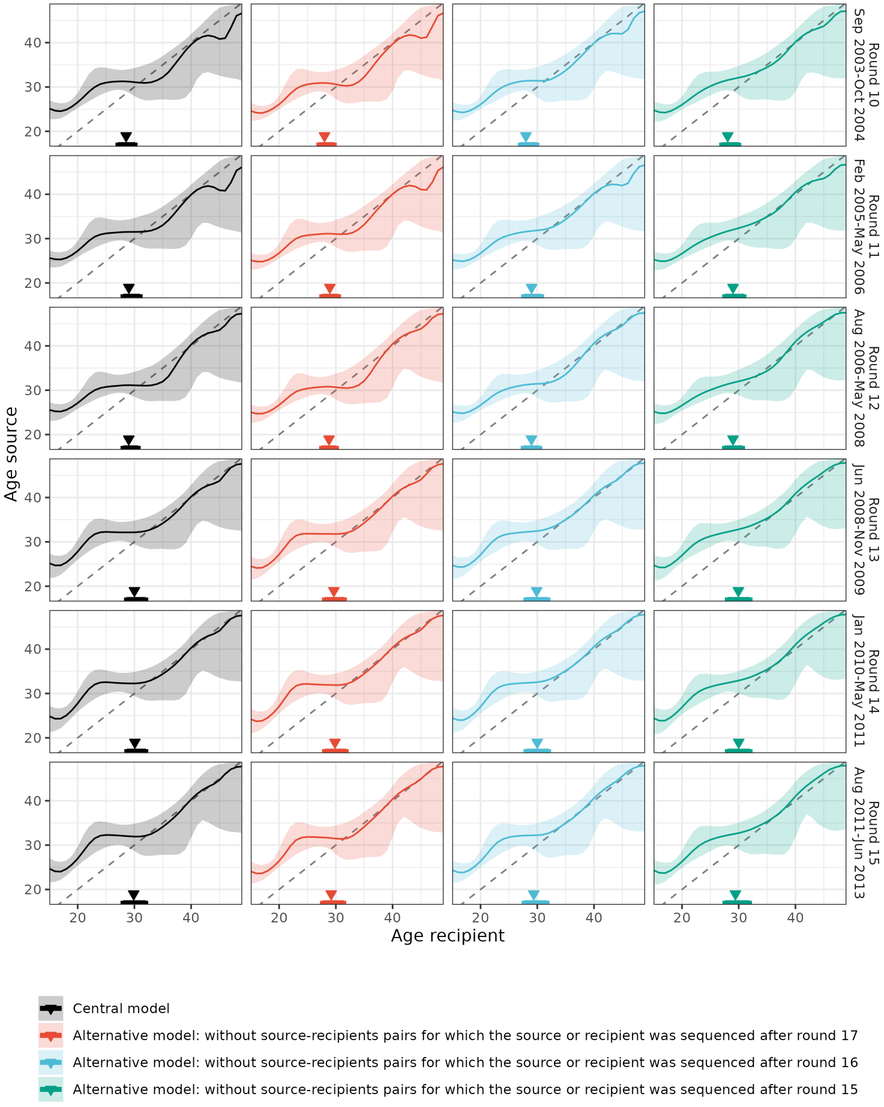

Supplementary Fig. S5: **Sensitivity in estimating the age of transmitting partners to right censoring of likely transmission pairs.** Posterior median (line) and 95% credible interval (ribbon) of the age of male transmitting partners by the age of the infected female (x-axis) by survey round (row facet) for the central and sensitivity analyses (column facet). Median and 95% credible interval of the age of male transmitting partners across the age of the infected female is indicated with a triangle and an error bar.

S2 Supplementary Tables

	Census-eligible individuals	Participants	Participants with HIV	Participants with HIV and with measured viral load	Participants with HIV reporting to be ART naïve†	Participants with HIV and with unsuppressed virus†	Participants with HIV and with virus ever deep-sequenced‡
**Round 10, September 26, 2003 - November 23, 2004; 28 communities surveyed**							
Total	11,976	7,407	884	–	–	–	115
Female	6,299	4,341	575	–	–	–	60
Age							
15-24	3,118	1,768	131	–	–	–	17
25-34	1,916	1,538	280	–	–	–	27
35-49	1,265	1,035	164	–	–	–	16
Male	5,677	3,066	309	–	–	–	55
Age							
15-24	2,672	1,186	38	–	–	–	9
25-34	1,845	1,132	145	–	–	–	27
35-49	1,160	748	126	–	–	–	19
**Round 11, February 15, 2005 - June 30, 2006; 28 communities surveyed**							
Total	12,528	8,273	1,002	884	–	–	176
Female	6,644	4,786	658	568	–	–	97
Age							
15-24	3,146	1,818	141	138	–	–	26
25-34	2,175	1,842	323	286	–	–	50
35-49	1,323	1,126	194	144	–	–	21
Male	5,884	3,487	344	316	–	–	79
Age							
15-24	2,670	1,293	30	30	–	–	6
25-34	1,956	1,290	160	153	–	–	40
35-49	1,258	904	154	133	–	–	33
**Round 12, August 30, 2006 - June 06, 2008; 28 communities surveyed**							
Total	13,718	8,752	1,105	912	–	–	234
Female	7,185	5,047	746	610	–	–	140
Age							
15-24	3,331	1,903	151	149	–	–	37
25-34	2,416	1,958	354	297	–	–	67
35-49	1,438	1,186	241	164	–	–	36
Male	6,533	3,705	359	302	–	–	94
Age							
15-24	2,866	1,426	26	25	–	–	8
25-34	2,200	1,305	168	156	–	–	50
35-49	1,467	974	165	121	–	–	36
**Round 13, June 17, 2008 - July 12, 2009; 28 communities surveyed**							
Total	13,433	8,718	1,160	900	–	–	369
Female	7,086	4,975	760	580	–	–	204
Age							
15-24	3,160	1,736	128	124	–	–	45
25-34	2,379	1,946	347	278	–	–	99
35-49	1,547	1,293	285	178	–	–	60
Male	6,347	3,743	400	320	–	–	165
Age							
15-24	2,749	1,397	32	31	–	–	19
25-34	2,042	1,275	177	160	–	–	82
35-49	1,556	1,071	191	129	–	–	64
**Round 14, January 18, 2010 - June 21, 2011; 28 communities surveyed**							
Total	14,828	9,663	1,313	964	–	–	602
Female	7,766	5,430	869	615	–	–	341
Age							
15-24	3,376	1,877	134	125	–	–	71
25-34	2,633	2,084	379	290	–	–	167
35-49	1,757	1,469	356	200	–	–	103
Male	7,062	4,233	444	349	–	–	261
Age							
15-24	2,963	1,617	40	38	–	–	31
25-34	2,276	1,398	185	163	–	–	120
35-49	1,823	1,218	219	148	–	–	110

† Unsuppressed virus was defined as a plasma viral load measurement above 1000 copies/mL plasma blood. In R10, participants were not asked about ART status and viral loads were not measured. In R11-R14, participants reported their ART status and viral loads were not measured. In R15, participants reported both their ART status and a subset of viral loads were measured. In R16-R18, participants reported both their ART status and viral loads were measured comprehensively in participants with HIV. ‡ Samples were selected for deep-sequencing from participants who had no viral load measured and reported being ART-naive or participants with viral load above 1,000 copies/mL plasma. Individuals participated across rounds, so for individuals participating in a given round, samples for sequencing could also be obtained in other rounds and we tabulate the proportion of participants ever deep-sequenced. Individuals with virus ever deep-sequenced were defined as HIV-positive individuals with deep-sequence output meeting minimum quality criteria, see Methods.

Supplementary Table S1: Characteristics of the RCCS study population.

S12 LIST OF TABLES

	**Census-eligible individuals**	**Participants**	**Participants with HIV**	**Participants with HIV and with measured viral load**	**Participants with HIV reporting to be ART naïve†**	**Participants with HIV and with unsuppressed virus†**	**Participants with HIV and with virus ever deep-sequenced‡**
**Round 15, August 10, 2011 - July 05, 2013; 33 communities surveyed**							
Total	20,806	13,589	1,944	1,331	207	367	1,086
Female	10,782	7,538	1,287	844	122	232	637
Age							
15-24	4,751	2,742	217	186	23	31	157
25-34	3,631	2,825	568	405	64	101	307
35-49	2,400	1,971	502	253	35	100	173
Male	10,024	6,051	657	487	85	135	449
Age							
15-24	4,150	2,368	68	58	10	11	54
25-34	3,243	1,955	260	218	41	57	208
35-49	2,631	1,728	329	211	34	67	187
**Round 16, July 08, 2013 - January 30, 2015; 35 communities surveyed**							
Total	21,887	14,072	1,875	868	671	1,829	893
Female	11,346	7,816	1,255	537	390	1,224	521
Age							
15-24	5,089	2,891	194	129	97	189	83
25-34	3,547	2,669	502	238	175	486	249
35-49	2,710	2,256	559	170	118	549	189
Male	10,541	6,256	620	331	281	605	372
Age							
15-24	4,436	2,462	50	40	34	47	35
25-34	3,241	1,883	219	141	123	212	155
35-49	2,864	1,911	351	150	124	346	182
**Round 17, February 23, 2015 - September 02, 2016; 35 communities surveyed**							
Total	22,929	15,093	2,015	646	514	2,004	934
Female	11,990	8,377	1,390	408	304	1,384	554
Age							
15-24	5,393	3,035	205	94	84	204	97
25-34	3,544	2,723	529	194	147	525	250
35-49	3,053	2,619	656	120	73	655	207
Male	10,939	6,716	625	238	210	620	380
Age							
15-24	4,677	2,662	41	28	26	40	31
25-34	3,121	1,912	208	102	91	206	139
35-49	3,141	2,142	376	108	93	374	210
**Round 18, October 03, 2016 - May 22, 2018; 35 communities surveyed**							
Total	23,269	15,053	1,860	432	375	1,850	849
Female	12,193	8,331	1,275	263	206	1,271	492
Age							
15-24	5,484	3,049	158	72	63	158	80
25-34	3,472	2,592	461	117	95	457	208
35-49	3,237	2,690	656	74	48	656	204
Male	11,076	6,722	585	169	169	579	357
Age							
15-24	4,739	2,671	38	22	24	36	27
25-34	3,077	1,850	183	79	78	183	128
35-49	3,260	2,201	364	68	67	360	202

† Unsuppressed virus was defined as a plasma viral load measurement above 1000 copies/mL plasma blood. In R10, participants were not asked about ART status and viral loads were not measured. In R11-R14, participants reported their ART status and viral loads were not measured. In R15, participants reported both their ART status and a subset of viral loads were measured. In R16-R18, participants reported both their ART status and viral loads were measured comprehensively in participants with HIV. ‡ Samples were selected for deep-sequencing from participants who had no viral load measured and reported being ART-naive or participants with viral load above 1,000 copies/mL plasma. Individuals participated across rounds, so for individuals participating in a given round, samples for sequencing could also be obtained in other rounds and we tabulate the proportion of participants ever deep-sequenced. Individuals with virus ever deep-sequenced were defined as HIV-positive individuals with deep-sequence output meeting minimum quality criteria, see Methods.

Supplementary Table S1: **Characteristics of the RCCS study population (continued).**

Community Identifier†	Part of RCCS								
	Round 10	Round 11	Round 12	Round 13	Round 14	Round 15	Round 16	Round 17	Round 18
i-01	Yes	Yes	Yes	Yes	Yes	Yes	Yes	Yes	Yes
i-02	Yes	Yes	Yes	Yes	Yes	Yes	Yes	Yes	Yes
i-03	Yes	Yes	Yes	Yes	Yes	Yes	Yes	Yes	Yes
i-04	Yes	Yes	Yes	Yes	Yes	Yes	Yes	Yes	Yes
i-05	Yes	Yes	Yes	Yes	Yes	Yes	Yes	Yes	Yes
i-06	Yes	Yes	Yes	Yes	Yes	Yes	Yes	Yes	Yes
i-07	Yes	Yes	Yes	Yes	Yes	Yes	Yes	Yes	Yes
i-08	Yes	Yes	Yes	Yes	Yes	Yes	Yes	Yes	Yes
i-09	No	No	No	No	No	Yes	Yes	Yes	Yes
i-10	Yes	Yes	Yes	Yes	Yes	Yes	Yes	Yes	Yes
i-11	Yes	Yes	Yes	Yes	Yes	Yes	Yes	Yes	Yes
i-12	Yes	Yes	Yes	Yes	Yes	Yes	Yes	Yes	Yes
i-13	No	No	No	No	No	No	Yes	Yes	Yes
i-14	No	No	No	No	No	Yes	Yes	Yes	Yes
i-15	Yes	Yes	Yes	Yes	Yes	Yes	Yes	Yes	Yes
i-16	Yes	Yes	Yes	Yes	Yes	Yes	Yes	Yes	Yes
i-17	Yes	Yes	Yes	Yes	Yes	Yes	Yes	Yes	Yes
i-18	No	No	No	No	No	No	Yes	Yes	Yes
i-19	Yes	Yes	Yes	Yes	Yes	Yes	Yes	Yes	Yes
i-20	No	No	No	No	No	Yes	No	No	No
i-21	No	No	No	No	No	No	Yes	Yes	Yes
i-22	Yes	Yes	Yes	Yes	Yes	Yes	Yes	Yes	Yes
i-23	Yes	Yes	Yes	Yes	Yes	Yes	Yes	Yes	Yes
i-24	Yes	Yes	Yes	Yes	Yes	Yes	Yes	Yes	Yes
i-25	Yes	Yes	Yes	Yes	Yes	Yes	Yes	Yes	Yes
i-26	Yes	Yes	Yes	Yes	Yes	Yes	Yes	Yes	Yes
i-27	Yes	Yes	Yes	Yes	Yes	Yes	Yes	Yes	Yes
i-28	Yes	Yes	Yes	Yes	Yes	Yes	Yes	Yes	Yes
i-29	Yes	Yes	Yes	Yes	Yes	Yes	Yes	Yes	Yes
i-30	Yes	Yes	Yes	Yes	Yes	Yes	Yes	Yes	Yes
i-31	Yes	Yes	Yes	Yes	Yes	Yes	Yes	Yes	Yes
i-32	No	No	No	No	No	Yes	Yes	Yes	Yes
i-33	No	No	No	No	No	Yes	Yes	Yes	Yes
i-34	Yes	Yes	Yes	Yes	Yes	Yes	Yes	Yes	Yes
i-35	Yes	Yes	Yes	Yes	Yes	Yes	Yes	Yes	Yes
i-36	Yes	Yes	Yes	Yes	Yes	Yes	Yes	Yes	Yes

† Three pairs of geographically close areas in peri-urban settings were merged into three communities.

Supplementary Table S2: Communities surveyed by RCCS in rounds 10-18.

S14 LIST OF TABLES

	Incidence cohort†	Person-years‡	Incidence events§	Incidence rate estimate per 100 PY¶
**Round 10, September 26, 2003 - November 23, 2004; 28 communities surveyed**				
Total	7,372	9,464.33 [9,448.40-9,481.02]	122.0 [112.22-130.77]	1.32 [1.27-1.37]
Female	4,055	5,213.53 [5,201.59-5,224.77]	71.0 [61.22-77.00]	1.37 [1.30-1.45]
Age				
15-24	1,706	1,938.12 [1,928.82-1,944.71]	32.0 [28.22-37.77]	1.53 [1.40-1.68]
25-34	1,440	2,025.25 [2,015.02-2,032.45]	26.0 [21.00-34.77]	1.50 [1.38-1.63]
35-49	909	1,251.31 [1,247.69-1,255.27]	11.0 [8.00-13.77]	0.90 [0.81-1.01]
Male	3,317	4,252.08 [4,237.69-4,264.78]	51.0 [45.22-57.32]	1.26 [1.19-1.33]
Age				
15-24	1,328	1,522.78 [1,514.43-1,527.77]	12.0 [7.22-14.77]	1.04 [0.94-1.15]
25-34	1,254	1,718.06 [1,708.34-1,725.71]	29.0 [24.23-34.77]	1.61 [1.48-1.75]
35-49	735	1,011.40 [1,006.45-1,015.40]	10.0 [8.00-13.00]	1.00 [0.89-1.11]
**Round 11, February 15, 2005 - June 30, 2006; 28 communities surveyed**				
Total	7,787	11,484.46 [11,465.55-11,505.89]	144.0 [131.45-154.77]	1.29 [1.23-1.34]
Female	4,291	6,261.90 [6,247.27-6,278.78]	84.0 [76.22-91.00]	1.36 [1.27-1.44]
Age				
15-24	1,646	2,088.35 [2,078.11-2,095.10]	31.0 [25.45-37.00]	1.48 [1.34-1.64]
25-34	1,667	2,654.38 [2,644.12-2,664.53]	39.0 [34.00-43.77]	1.46 [1.34-1.59]
35-49	978	1,519.83 [1,515.14-1,526.38]	13.0 [11.00-17.00]	1.00 [0.90-1.11]
Male	3,496	5,222.93 [5,209.14-5,243.01]	60.0 [51.45-65.00]	1.20 [1.14-1.27]
Age				
15-24	1,323	1,781.07 [1,774.24-1,787.04]	17.0 [12.00-20.00]	0.97 [0.88-1.06]
25-34	1,356	2,145.73 [2,135.78-2,156.34]	31.0 [26.23-36.77]	1.55 [1.43-1.69]
35-49	817	1,296.59 [1,291.00-1,302.85]	11.0 [8.00-14.77]	0.95 [0.86-1.06]
**Round 12, August 30, 2006 - June 06, 2008; 28 communities surveyed**				
Total	8,480	12,396.23 [12,369.28-12,422.54]	168.0 [151.12-177.33]	1.21 [1.16-1.28]
Female	4,598	6,648.49 [6,632.13-6,668.15]	95.0 [84.67-101.00]	1.31 [1.24-1.43]
Age				
15-24	1,669	2,100.25 [2,091.86-2,108.29]	31.0 [25.00-36.77]	1.44 [1.29-1.62]
25-34	1,869	2,883.98 [2,869.79-2,897.39]	45.0 [39.23-52.77]	1.39 [1.27-1.54]
35-49	1,060	1,666.57 [1,659.44-1,673.22]	19.0 [16.00-22.00]	1.02 [0.90-1.18]
Male	3,882	5,746.59 [5,732.78-5,759.56]	72.0 [65.22-79.00]	1.09 [1.02-1.17]
Age				
15-24	1,460	1,990.10 [1,984.61-1,996.47]	15.0 [10.22-17.00]	0.83 [0.75-0.92]
25-34	1,474	2,246.22 [2,235.56-2,252.48]	38.0 [32.00-44.00]	1.46 [1.34-1.59]
35-49	948	1,511.44 [1,503.26-1,516.12]	19.0 [16.23-23.77]	0.88 [0.80-1.00]
**Round 13, June 17, 2008 - July 12, 2009; 28 communities surveyed**				
Total	8,770	11,823.39 [11,802.83-11,845.07]	136.0 [125.00-145.55]	1.08 [1.04-1.15]
Female	4,728	6,331.90 [6,313.25-6,348.15]	83.0 [73.45-89.00]	1.21 [1.15-1.33]
Age				
15-24	1,624	1,942.24 [1,932.52-1,949.30]	29.0 [25.00-35.77]	1.38 [1.26-1.54]
25-34	1,948	2,723.50 [2,708.26-2,732.27]	37.0 [32.00-43.55]	1.27 [1.17-1.41]
35-49	1,156	1,667.46 [1,661.12-1,673.50]	16.0 [12.00-21.77]	0.90 [0.81-1.06]
Male	4,042	5,490.33 [5,477.08-5,500.21]	52.0 [47.23-59.55]	0.94 [0.89-1.02]
Age				
15-24	1,491	1,900.09 [1,893.07-1,905.26]	17.0 [13.23-21.55]	0.69 [0.63-0.77]
25-34	1,475	2,004.64 [1,996.54-2,012.04]	23.0 [18.00-27.77]	1.30 [1.20-1.43]
35-49	1,076	1,586.25 [1,578.36-1,593.72]	13.0 [10.00-16.77]	0.78 [0.70-0.89]
**Round 14, January 18, 2010 - June 21, 2011; 28 communities surveyed**				
Total	9,290	12,359.17 [12,344.41-12,374.39]	107.5 [97.45-118.00]	0.93 [0.89-0.97]
Female	4,963	6,624.63 [6,608.63-6,638.01]	63.0 [55.00-71.78]	1.07 [1.00-1.13]
Age				
15-24	1,706	1,998.64 [1,991.11-2,007.00]	23.0 [19.00-30.00]	1.30 [1.17-1.43]
25-34	1,999	2,766.97 [2,761.12-2,775.18]	24.0 [15.68-30.55]	1.13 [1.03-1.22]
35-49	1,258	1,857.69 [1,850.32-1,863.26]	15.0 [11.00-19.00]	0.74 [0.66-0.82]
Male	4,327	5,734.81 [5,725.76-5,744.10]	46.0 [39.23-50.00]	0.77 [0.73-0.82]
Age				
15-24	1,642	1,988.89 [1,983.58-1,992.83]	14.0 [10.00-16.77]	0.55 [0.50-0.61]
25-34	1,487	1,999.30 [1,992.87-2,005.68]	22.0 [19.00-26.77]	1.11 [1.02-1.21]
35-49	1,198	1,747.16 [1,742.21-1,752.55]	9.0 [6.22-12.77]	0.65 [0.58-0.73]

† Number of RCCS study participants who were HIV-negative at their first visit and had at least one subsequent follow-up visit. ‡ Number of person-years of HIV acquisition risk. § Number of incidence events. The infection date was imputed at random to have occurred between the last negative and first positive survey visit dates, and the incidence event was attributed to the corresponding survey round 50 times. The range of the person-years and incidence events across the 50 data sets with imputed exposure times are presented. ¶ Estimated incidence rate per 100 person-years. The confidence interval of the estimated incidence rate incorporates both the variability of the estimation procedure and the data imputation procedure.

Supplementary Table S3: **Characteristics of the longitudinal HIV incidence cohort.**

	Incidence cohort†	Person-years‡	Incidence events§	Incidence rate estimate per 100 PY¶
**Round 15, August 10, 2011 - July 05, 2013; 33 communities surveyed**				
Total	10,441	17,621.81 [17,596.06-17,643.04]	140.0 [129.45-148.78]	0.79 [0.76-0.83]
Female	5,520	9,227.87 [9,204.36-9,242.47]	87.0 [79.22-94.77]	0.94 [0.88-0.99]
Age				
15-24	1,892	2,742.21 [2,728.96-2,752.62]	37.0 [31.23-43.77]	1.17 [1.05-1.30]
25-34	2,184	3,728.50 [3,713.89-3,735.50]	38.0 [34.00-42.77]	1.02 [0.92-1.10]
35-49	1,444	2,757.15 [2,750.51-2,765.25]	12.0 [9.23-15.77]	0.61 [0.54-0.68]
Male	4,921	8,395.89 [8,383.12-8,406.96]	52.0 [47.23-60.00]	0.64 [0.60-0.67]
Age				
15-24	1,848	2,842.07 [2,836.70-2,847.92]	11.0 [8.00-14.00]	0.45 [0.41-0.50]
25-34	1,657	2,865.12 [2,856.30-2,874.43]	31.0 [26.23-35.00]	0.92 [0.84-1.01]
35-49	1,416	2,687.98 [2,679.81-2,695.84]	11.0 [6.22-14.00]	0.52 [0.46-0.59]
**Round 16, July 08, 2013 - January 30, 2015; 35 communities surveyed**				
Total	12,142	16,633.57 [16,621.16-16,648.28]	108.5 [98.45-116.78]	0.66 [0.63-0.70]
Female	6,380	8,745.06 [8,737.02-8,758.26]	72.5 [64.22-80.78]	0.80 [0.75-0.86]
Age				
15-24	2,236	2,699.66 [2,693.50-2,703.90]	24.5 [21.23-31.55]	0.89 [0.80-0.99]
25-34	2,328	3,202.15 [3,195.15-3,209.33]	33.0 [27.00-38.77]	0.94 [0.85-1.04]
35-49	1,816	2,843.90 [2,839.95-2,847.65]	15.0 [11.22-18.00]	0.55 [0.49-0.62]
Male	5,762	7,888.21 [7,881.14-7,895.54]	35.0 [31.00-39.00]	0.51 [0.48-0.55]
Age				
15-24	2,206	2,803.63 [2,801.36-2,806.94]	8.0 [7.00-10.00]	0.37 [0.32-0.41]
25-34	1,813	2,501.71 [2,496.99-2,507.33]	17.0 [13.00-20.00]	0.77 [0.68-0.84]
35-49	1,743	2,582.08 [2,578.79-2,588.18]	9.0 [6.22-14.00]	0.43 [0.37-0.49]
**Round 17, February 23, 2015 - September 02, 2016; 35 communities surveyed**				
Total	12,738	17,437.70 [17,422.40-17,448.35]	89.5 [80.22-95.78]	0.56 [0.53-0.59]
Female	6,680	9,116.75 [9,106.85-9,127.51]	57.0 [48.45-61.77]	0.68 [0.64-0.72]
Age				
15-24	2,327	2,796.00 [2,790.86-2,799.37]	11.0 [8.00-13.77]	0.62 [0.56-0.70]
25-34	2,286	3,187.45 [3,182.16-3,194.41]	28.0 [23.23-32.00]	0.87 [0.80-0.95]
35-49	2,067	3,133.05 [3,127.18-3,138.08]	17.0 [15.00-21.77]	0.53 [0.48-0.59]
Male	6,058	8,321.01 [8,312.47-8,328.62]	32.0 [27.45-36.00]	0.43 [0.40-0.46]
Age				
15-24	2,353	3,012.95 [3,009.30-3,015.97]	9.0 [8.00-11.00]	0.30 [0.27-0.35]
25-34	1,796	2,485.06 [2,479.65-2,490.25]	14.0 [10.22-18.00]	0.65 [0.58-0.73]
35-49	1,909	2,823.11 [2,818.49-2,830.23]	9.0 [5.22-12.00]	0.36 [0.30-0.42]
**Round 18, October 03, 2016 - May 22, 2018; 35 communities surveyed**				
Total	12,217	17,992.52 [17,982.46-18,005.50]	89.0 [83.00-97.78]	0.50 [0.47-0.54]
Female	6,425	9,624.65 [9,617.33-9,633.49]	57.0 [53.00-65.00]	0.62 [0.56-0.68]
Age				
15-24	2,174	2,703.74 [2,699.61-2,706.79]	12.0 [10.00-13.77]	0.42 [0.35-0.51]
25-34	2,125	3,249.56 [3,241.74-3,255.03]	26.0 [24.00-30.77]	0.85 [0.75-0.96]
35-49	2,126	3,671.67 [3,665.44-3,676.22]	19.0 [16.23-23.00]	0.56 [0.47-0.65]
Male	5,792	8,368.03 [8,361.41-8,377.69]	32.0 [30.00-35.00]	0.37 [0.34-0.40]
Age				
15-24	2,229	2,895.16 [2,891.38-2,899.31]	10.0 [8.00-12.00]	0.26 [0.22-0.31]
25-34	1,664	2,496.56 [2,493.55-2,501.84]	14.0 [12.00-17.00]	0.56 [0.49-0.64]
35-49	1,899	2,976.37 [2,972.27-2,980.31]	8.0 [6.00-11.00]	0.31 [0.25-0.37]

† Number of RCCS study participants who were HIV-negative at their first visit and had at least one subsequent follow-up visit. ‡ Number of person-years of HIV acquisition risk. § Number of incidence events. The infection date was imputed at random to have occurred between the last negative and first positive survey visit dates, and the incidence event was attributed to the corresponding survey round 50 times. The range of the person-years and incidence events across the 50 data sets with imputed exposure times are presented. ¶ Estimated incidence rate per 100 person-years. The confidence interval of the estimated incidence rate incorporates both the variability of the estimation procedure and the data imputation procedure.

Supplementary Table S3: Characteristics of the longitudinal HIV incidence cohort (continued).

S16 LIST OF TABLES

	Akaike information criterion (AIC) Men	Women	% observations within 95% prediction intervals Men	Women	All
Central model					
	**8,032** [7,937-8,140]	**11,579** [11,508-11,688]	98.77% [97.78-99.68]	**98.82%** [97.78-99.68]	**98.80%** [98.10-99.49]
Alternative models					
with 2D GP over age and survey round	8,033 [7,938-8,141]	11,580 [11,511-11,690]	**98.84%** [98.10-99.68]	93.32% [91.18-95.10]	96.08% [94.96-96.95]
without interaction term between age and survey round	8,033 [7,938-8,142]	11,592 [11,521-11,706]	98.79% [97.78-99.68]	93.83% [92.06-95.24]	96.31% [95.27-97.23]
with 2D GP over age and survey round and without interaction term between age and survey round	8,035 [7,939-8,143]	11,590 [11,517-11,701]	98.82% [97.78-99.68]	93.45% [90.94-95.24]	96.13% [94.99-97.23]

Supplementary Table S4: **Model comparison for estimating longitudinal, age-specific incidence rates.**

	Participants with HIV (n)	Participants with HIV >1,000 cps/mL or or reporting no ART use if viral load was not measured (n)	Participants with HIV and with virus ever deep-sequenced with Illumina MiSeq in PANGEA-HIV 1 † (n)	Participants with HIV and with virus ever deep-sequenced with Illumina HiSeq in PANGEA-HIV 1 ‡ (n)	Participants with HIV and with virus ever deep-sequenced with Illumina NovaSeq in PANGEA-HIV 2 § (n)	Participants with HIV and with virus ever deep-sequenced (n)	Sequence sampling coverage of participants with HIV (%)
**Round 10, September 26, 2003 - November 23, 2004; 28 communities surveyed**							
Total	884	884	54	3	58	115	13.01
Female	575	575	25	2	33	60	10.43
Age							
15-24	131	131	8	1	8	17	12.98
25-34	280	280	9	0	18	27	9.64
35-49	164	164	8	1	7	16	9.76
Male	309	309	29	1	25	55	17.8
Age							
15-24	38	38	6	0	3	9	23.68
25-34	145	145	12	1	14	27	18.62
35-49	126	126	11	0	8	19	15.08
**Round 11, February 15, 2005 - June 30, 2006; 28 communities surveyed**							
Total	1002	884	80	3	93	176	17.56
Female	658	568	41	2	54	97	14.74
Age							
15-24	141	138	8	1	17	26	18.44
25-34	323	286	22	0	28	50	15.48
35-49	194	144	11	1	9	21	10.82
Male	344	316	39	1	39	79	22.97
Age							
15-24	30	30	4	0	2	6	20
25-34	160	153	20	1	19	40	25
35-49	154	133	15	0	18	33	21.43
**Round 12, August 30, 2006 - June 06, 2008; 28 communities surveyed**							
Total	1105	912	117	3	114	234	21.18
Female	746	610	63	2	75	140	18.77
Age							
15-24	151	149	16	1	20	37	24.5
25-34	354	297	31	0	36	67	18.93
35-49	241	164	16	1	19	36	14.94
Male	359	302	54	1	39	94	26.18
Age							
15-24	26	25	6	0	2	8	30.77
25-34	168	156	28	1	21	50	29.76
35-49	165	121	20	0	16	36	21.82
**Round 13, June 17, 2008 - July 12, 2009; 28 communities surveyed**							
Total	1160	900	179	3	187	369	31.81
Female	760	580	93	2	109	204	26.84
Age							
15-24	128	124	22	1	22	45	35.16
25-34	347	278	44	0	55	99	28.53
35-49	285	178	27	1	32	60	21.05
Male	400	320	86	1	78	165	41.25
Age							
15-24	32	31	14	0	5	19	59.38
25-34	177	160	41	1	40	82	46.33
35-49	191	129	31	0	33	64	33.51
**Round 14, January 18, 2010 - June 21, 2011; 28 communities surveyed**							
Total	1313	964	305	3	294	602	45.85
Female	869	615	166	2	173	341	39.24
Age							
15-24	134	125	40	0	31	71	52.99
25-34	379	290	81	1	85	167	44.06
35-49	356	200	45	1	57	103	28.93
Male	444	349	139	1	121	261	58.78
Age							
15-24	40	38	20	0	11	31	77.5
25-34	185	163	58	1	61	120	64.86
35-49	219	148	61	0	49	110	50.23

† RNA samples were sequenced using the protocol of[1] at the Wellcome Trust Sanger Institute, Hinxton, UK on Illumina MiSeq platforms. Deep-sequences reported satisfied minimum quality criteria for deep-sequence phylogenetic analysis, see Methods. ‡ As for previous column, on Illumina HiSeq platforms. § RNA samples were sequenced using the protocol of[2] at the Oxford Genomics Centre, Oxford, UK on Illumina NovaSeq 6000 platforms. Deep-sequences reported satisfied minimum quality criteria for deep-sequence phylogenetic analysis, see Methods.

Supplementary Table S5: Longitudinal HIV deep-sequencing.

S18 LIST OF TABLES

	Participants with HIV (n)	Participants with HIV >1,000 cps/mL or reporting no ART use if viral load was not measured (n)	Participants with HIV and with virus ever deep-sequenced with Illumina MiSeq in PANGEA-HIV 1 † (n)	Participants with HIV and with virus ever deep-sequenced with Illumina HiSeq in PANGEA-HIV 1 ‡ (n)	Participants with HIV and with virus ever deep-sequenced with Illumina NovaSeq in PANGEA-HIV 2 § (n)	Participants with HIV and with virus ever deep-sequenced (n)	Sequence sampling coverage of participants with HIV (%)
**Round 15, August 10, 2012 - July 05, 2013; 33 communities surveyed**							
Total	1901	1298	282	2	802	1086	57.13
Female	1264	827	152	1	484	637	50.4
Age							
15-24	209	178	23	0	134	157	75.12
25-34	557	398	85	1	221	307	55.12
35-49	498	251	44	0	129	173	34.74
Male	637	471	130	1	318	449	70.49
Age							
15-24	67	57	17	0	37	54	80.6
25-34	249	208	55	0	153	208	83.53
35-49	321	206	58	1	128	187	58.26
**Round 16, July 08, 2013 - January 30, 2015; 35 communities surveyed**							
Total	1874	869	383	3	506	892	47.6
Female	1254	536	212	1	307	520	41.47
Age							
15-24	194	129	36	0	47	83	42.78
25-34	502	238	108	1	140	249	49.6
35-49	558	169	68	0	120	188	33.69
Male	620	333	171	2	199	372	60
Age							
15-24	50	40	21	0	14	35	70
25-34	219	141	75	0	80	155	70.78
35-49	351	152	75	2	105	182	51.85
**Round 17, February 23, 2015 - September 02, 2016; 35 communities surveyed**							
Total	2015	639	604	4	326	934	46.35
Female	1390	402	348	2	204	554	39.86
Age							
15-24	205	91	82	0	15	97	47.32
25-34	529	190	163	2	85	250	47.26
35-49	656	121	103	0	104	207	31.55
Male	625	237	256	2	122	380	60.8
Age							
15-24	41	28	28	0	3	31	75.61
25-34	208	102	101	0	38	139	66.83
35-49	376	107	127	2	81	210	55.85
**Round 18, October 03, 2016 - May 22, 2018; 35 communities surveyed**							
Total	1860	416	565	2	282	849	45.65
Female	1275	255	315	1	176	492	38.59
Age							
15-24	158	71	72	0	8	80	50.63
25-34	461	111	135	1	72	208	45.12
35-49	656	73	108	0	96	204	31.1
Male	585	161	250	1	106	357	61.03
Age							
15-24	38	22	26	0	1	27	71.05
25-34	183	76	101	0	27	128	69.95
35-49	364	63	123	1	78	202	55.49

† RNA samples were sequenced using the protocol of[1] at the Wellcome Trust Sanger Institute, Hinxton, UK on Illumina MiSeq platforms. Deep-sequences reported satisfied minimum quality criteria for deep-sequence phylogenetic analysis, see Methods. ‡ As for previous column, on Illumina HiSeq platforms. § RNA samples were sequenced using the protocol of[2] at the Oxford Genomics Centre, Oxford, UK on Illumina NovaSeq 6000 platforms. Deep-sequences reported satisfied minimum quality criteria for deep-sequence phylogenetic analysis, see Methods.

Supplementary Table S5: **Longitudinal HIV deep-sequencing (continued).**

	Observed transmission events within 95% prediction interval (%)	Observed transmission events vs. predicted transmission events (MAE)†	Incidence rate prior mean within 95% posterior range (%)	Incidence rate prior mean vs. incidence rate posterior median (MAE)†
**Central model**				
$\log \beta^{g\to h}_{r,i,j} = \hat{\boldsymbol{c}}^{g\to h}(i,j) + \gamma_0 + \gamma_g + \gamma_r + \gamma_{p(r)} + \boldsymbol{f}^{g\to h}_0(i,j) + \boldsymbol{f}^{g\to h}_r(j) + \boldsymbol{f}^{g\to h}_{p(r)}(i)$, (7c)	99.63	0.0459	97.14	0.00032
**Alternative models**				
$\log \beta^{g\to h}_{r,i,j} = \hat{\boldsymbol{c}}^{g\to h}(i,j) + \gamma_0 + \gamma_g + \gamma_r + \gamma_{p(r)} + \boldsymbol{f}^{g\to h}_0(i,j) + \boldsymbol{f}^{g\to h}_{p(r)}(i)$, (12a)	99.59	0.0473	67.78	0.00057
$\log \beta^{g\to h}_{r,i,j} = \hat{\boldsymbol{c}}^{g\to h}(i,j) + \gamma_0 + \gamma_g + \gamma_r + \gamma_{p(r)} + \boldsymbol{f}^{g\to h}_0(i,j) + \boldsymbol{f}^{g\to h}_{p(r)}(j)$, (12b)	99.61	0.0467	67.62	0.00058
$\log \beta^{g\to h}_{r,i,j} = \hat{\boldsymbol{c}}^{g\to h}(i,j) + \gamma_0 + \gamma_g + \gamma_r + \gamma_{p(r)} + \boldsymbol{f}^{g\to h}_0(i,j) + \boldsymbol{f}^{g\to h}_{p(r)}(i,j)$, (12c)	99.57	0.0471	68.89	0.00056
$\log \beta^{g\to h}_{r,i,j} = \hat{\boldsymbol{c}}^{g\to h}(i,j) + \gamma_0 + \gamma_g + \gamma_r + \gamma_{p(r)} + \boldsymbol{f}^{g\to h}_0(i,j) + \boldsymbol{f}^{g\to h}_r(j)$, (12d)	99.57	0.0457	96.35	0.00033
$\log \beta^{g\to h}_{r,i,j} = \hat{\boldsymbol{c}}^{g\to h}(i,j) + \gamma_0 + \gamma_g + \gamma_r + \gamma_{p(r)} + \boldsymbol{f}^{g\to h}_0(i,j) + \boldsymbol{f}^{g\to h}_r(j) + \boldsymbol{f}^{g\to h}_{p(r)}(j)$, (12e)	99.53	0.0459	97.94	0.00031
$\log \beta^{g\to h}_{r,i,j} = \hat{\boldsymbol{c}}^{g\to h}(i,j) + \gamma_0 + \gamma_g + \gamma_r + \gamma_{p(r)} + \boldsymbol{f}^{g\to h}_0(i,j) + \boldsymbol{f}^{g\to h}_r(j) + \boldsymbol{f}^{g\to h}_{p(r)}(i,j)$, (12f)	99.61	0.0459	97.14	0.00031

† MAE: Mean absolute error.

Supplementary Table S6: **Model comparison for estimating longitudinal, age-specific transmission flows.**

S20 LIST OF TABLES

Transmission direction	Male-female difference in age at transmission	Infected partner by age at transmission: 15-24 years (%)†	25-34 years (%)†	35-49 years (%)†	Total (%)†
**Round 10, September 26, 2003 - November 23, 2004; 28 communities surveyed**					
Male to female	Total	31.9% [30.2-33.5]	18.8% [17.9-19.7]	7.3% [6.7-7.9]	57.9% [56.2-59.6]
	<0 years	0.4% [0.2-0.6]	5.6% [3.9-7.4]	4.0% [2.6-5.5]	10.0% [7.5-12.5]
	0-6 years	15.5% [12.3-18.9]	7.7% [6.2-9.3]	3.0% [1.8-4.4]	26.3% [22.4-30.4]
	>6 years	16.0% [12.7-19.2]	5.4% [3.9-7.3]	0.2% [0.0-0.5]	21.6% [17.6-25.7]
Female to male	Total	14.8% [13.9-15.8]	20.6% [19.7-21.6]	6.6% [6.2-7.1]	42.1% [40.4-43.8]
	<0 years	6.6% [4.9-8.3]	4.7% [3.2-6.5]	0.4% [0.2-0.8]	11.7% [8.8-14.9]
	0-6 years	8.2% [6.2-10.1]	11.8% [9.9-13.4]	2.6% [1.8-3.4]	22.5% [19.4-25.6]
	>6 years	0.1% [0.0-0.2]	4.1% [2.7-5.9]	3.6% [2.6-4.7]	7.8% [5.7-10.1]
Total		46.7% [45.3-48.1]	39.4% [38.3-40.6]	13.9% [13.2-14.6]	100%
**Round 15, August 10, 2011 - July 05, 2013; 33 communities surveyed**					
Male to female	Total	32.2% [30.2-34.3]	22.0% [20.7-23.4]	7.7% [7.0-8.5]	61.9% [60.2-63.7]
	<0 years	0.5% [0.2-0.8]	6.0% [4.1-8.1]	3.8% [2.3-5.4]	10.3% [7.6-13.1]
	0-6 years	15.4% [12.2-19.0]	9.0% [7.1-11.0]	3.6% [2.2-5.0]	28.0% [23.8-32.4]
	>6 years	16.2% [12.8-19.7]	7.0% [5.1-9.1]	0.3% [0.1-0.8]	23.6% [19.2-28.0]
Female to male	Total	11.5% [10.6-12.4]	18.8% [17.8-19.9]	7.8% [7.2-8.4]	38.1% [36.3-39.8]
	<0 years	6.2% [4.8-7.7]	4.6% [3.2-6.4]	0.6% [0.2-1.1]	11.4% [8.8-14.3]
	0-6 years	5.2% [3.9-6.6]	11.3% [9.6-12.8]	3.2% [2.2-4.3]	19.7% [16.9-22.4]
	>6 years	0.0% [0.0-0.0]	2.9% [1.9-4.1]	3.9% [2.8-5.2]	6.9% [5.1-8.8]
Total		43.6% [41.8-45.5]	40.9% [39.3-42.4]	15.5% [14.5-16.4]	100%
**Round 18, October 03, 2016 - May 22, 2018; 35 communities surveyed**					
Male to female	Total	20.6% [18.1-23.4]	27.3% [25.2-29.5]	14.7% [13.3-16.3]	62.8% [60.2-65.2]
	<0 years	0.3% [0.1-0.6]	6.7% [3.9-10.1]	7.0% [4.5-9.7]	14.0% [9.8-18.8]
	0-6 years	8.1% [5.6-11.0]	12.0% [9.1-15.0]	7.1% [4.7-9.7]	27.3% [23.1-31.8]
	>6 years	12.1% [9.3-15.2]	8.5% [5.8-11.9]	0.5% [0.1-1.5]	21.3% [16.8-26.3]
Female to male	Total	11.2% [9.9-12.6]	17.4% [15.9-19.1]	8.6% [7.6-9.7]	37.2% [34.8-39.8]
	<0 years	5.5% [3.9-7.3]	3.8% [2.5-5.5]	0.5% [0.2-1.1]	9.8% [7.2-13.0]
	0-6 years	5.7% [3.9-7.4]	10.6% [8.9-12.3]	3.2% [2.0-4.5]	19.5% [16.6-22.4]
	>6 years	0.0% [0.0-0.1]	2.9% [1.9-4.3]	4.9% [3.5-6.5]	7.9% [5.9-10.1]
Total		31.8% [29.4-34.5]	44.8% [42.5-47.0]	23.4% [21.6-25.2]	100%

† Posterior median flow estimates and 95% credible intervals in each survey round.

Supplementary Table S7: Longitudinal HIV transmission flows by age and gender.

	**Participants** (n)	**Contacts with reported partner characteristics** (%)	**Reported contacts per participant** (n)	**Estimated contacts per person** (median, 95% CrI)	**Estimated reporting bias** (median, 95% CrI)	**Reported contacts scaled to population** (n)	**Estimated contacts scaled to population** (median, 95% CrI)
Total	13,277	85.1	0.74	0.84 [0.76, 0.95]	0.1 [0.02, 0.21]	16,025	18,183 [16,450, 20,613]
Female	7,375	87.69	0.64	0.81 [0.74, 0.91]	0.17 [0.10, 0.27]	7,189	9,092 [8,284, 10,238]
Age							
15-19	1,296	84.20	0.34	0.48 [0.44, 0.54]	0.14 [0.09, 0.20]	844	1,187 [1,067, 1,321]
20-24	1,378	91.06	0.84	1.17 [1.09, 1.25]	0.33 [0.25, 0.41]	1,787	2,487 [2,324, 2,662]
25-29	1,432	85.99	0.90	1.18 [1.10, 1.26]	0.27 [0.20, 0.36]	1,704	2,221 [2,074, 2,381]
30-34	1,323	87.64	0.84	0.99 [0.92, 1.08]	0.15 [0.07, 0.24]	1,334	1,569 [1,451, 1,705]
35-39	1,007	87.60	0.75	0.83 [0.75, 0.95]	0.08 [0.00, 0.20]	849	942 [847, 1,075]
40-44	562	90.03	0.60	0.65 [0.55, 0.81]	0.05 [-0.05, 0.21]	436	472 [398, 588]
45-49	377	83.73	0.49	0.34 [0.21, 0.61]	-0.15 [-0.28, 0.12]	236	164 [102, 293]
50-54	0	-	-	0.13 [0.06, 0.36]	- -	-	43 [20, 124]
55-59	0	-	-	0.01 [0.00, 0.18]	- -	-	4 [1, 45]
60-64	0	-	-	0.01 [0.00, 0.14]	- -	-	1 [0, 24]
65-69	0	-	-	0.01 [0.00, 0.17]	- -	-	1 [0, 20]
Male	5,902	82.58	0.85	0.88 [0.79, 1.00]	0.02 [-0.06, 0.15]	8,836	9,091 [8,166, 10,374]
Age							
15-19	1,295	66.42	0.20	0.17 [0.14, 0.20]	-0.04 [-0.06, -0.01]	444	363 [306, 431]
20-24	1,001	75.50	0.84	0.79 [0.72, 0.87]	-0.04 [-0.11, 0.03]	1,528	1,447 [1,321, 1,585]
25-29	1,001	82.29	1.17	1.15 [1.07, 1.24]	-0.02 [-0.10, 0.07]	1,928	1,902 [1,763, 2,049]
30-34	913	84.05	1.26	1.28 [1.19, 1.37]	0.02 [-0.08, 0.11]	1,858	1,881 [1,747, 2,022]
35-39	796	83.82	1.36	1.31 [1.21, 1.41]	-0.05 [-0.14, 0.05]	1,587	1,530 [1,418, 1,648]
40-44	554	88.94	1.20	1.22 [1.11, 1.33]	0.01 [-0.09, 0.12]	990	999 [913, 1,089]
45-49	342	91.35	0.97	1.12 [0.98, 1.27]	0.15 [0.01, 0.30]	502	580 [509, 656]
50-54	0	-	-	0.79 [0.47, 1.31]	- -	-	251 [151, 417]
55-59	0	-	-	0.48 [0.15, 1.43]	- -	-	98 [30, 290]
60-64	0	-	-	0.26 [0.06, 1.14]	- -	-	33 [7, 142]
65-69	0	-	-	0.10 [0.02, 0.62]	- -	-	7 [1, 45]

Supplementary Table S8: **Sexual behaviour characteristics in RCCS participants, round 15, October 08 2011 - July 05 2013.**

	Participants reporting no ART use and who have suppressed virus	Participants reporting no ART use and who have unsuppressed virus	Participants reporting ART use and who have suppressed virus	Participants reporting ART use and who have unsuppressed virus	Sensitivity	Specificity
**Round 15, August 10, 2011 - July 05, 2013; 33 communities surveyed**						
Total	65	202	95	5	95.0% [88.5- 98.1]	75.7% [70.2- 80.4]
Female	44	118	66	4	94.3% [85.8- 98.2]	72.8% [65.5- 79.1]
Age						
15-24	5	22	3	1	75.0% [28.9- 96.6]	81.5% [62.8- 92.3]
25-34	19	63	18	1	94.7% [73.5-100.0]	76.8% [66.5- 84.7]
35-49	20	33	45	2	95.7% [85.0- 99.6]	62.3% [48.8- 74.1]
Male	21	84	29	1	96.7% [81.9-100.0]	80.0% [71.3- 86.6]
Age						
15-24	1	10	0	0		90.9% [60.1-100.0]
25-34	8	41	8	0	100.0% [62.8-100.0]	83.7% [70.7- 91.8]
35-49	12	33	21	1	95.5% [76.5-100.0]	73.3% [58.8- 84.2]
**Round 16, July 08, 2013 - January 30, 2015; 35 communities surveyed**						
Total	235	596	923	75	92.5% [90.7- 94.0]	71.7% [68.6- 74.7]
Female	171	342	663	48	93.2% [91.1- 94.9]	66.7% [62.5- 70.6]
Age						
15-24	37	87	55	10	84.6% [73.7- 91.6]	70.2% [61.6- 77.5]
25-34	72	152	239	23	91.2% [87.1- 94.1]	67.9% [61.5- 73.6]
35-49	62	103	369	15	96.1% [93.6- 97.7]	62.4% [54.8- 69.5]
Male	64	254	260	27	90.6% [86.6- 93.5]	79.9% [75.1- 83.9]
Age						
15-24	5	32	8	2	80.0% [47.9- 95.4]	86.5% [71.5- 94.6]
25-34	19	115	70	8	89.7% [80.8- 94.9]	85.8% [78.8- 90.8]
35-49	40	107	182	17	91.5% [86.7- 94.7]	72.8% [65.1- 79.4]
**Round 17, February 23, 2015 - September 02, 2016; 35 communities surveyed**						
Total	221	421	1269	93	93.2% [91.7- 94.4]	65.6% [61.8- 69.2]
Female	165	241	915	63	93.6% [91.8- 94.9]	59.4% [54.5- 64.0]
Age						
15-24	28	66	92	18	83.6% [75.5- 89.5]	70.2% [60.3- 78.5]
25-34	73	119	305	28	91.6% [88.1- 94.2]	62.0% [54.9- 68.6]
35-49	64	56	518	17	96.8% [94.9- 98.0]	46.7% [38.0- 55.6]
Male	56	180	354	30	92.2% [89.0- 94.5]	76.3% [70.4- 81.3]
Age						
15-24	3	24	11	2	84.6% [56.5- 96.9]	88.9% [71.1- 97.0]
25-34	19	82	96	9	91.4% [84.3- 95.6]	81.2% [72.4- 87.7]
35-49	34	74	247	19	92.9% [89.1- 95.4]	68.5% [59.2- 76.5]
**Round 18, October 03, 2016 - May 22, 2018; 35 communities surveyed**						
Total	141	288	1334	87	93.9% [92.5- 95.0]	67.1% [62.6- 71.4]
Female	109	153	956	53	94.7% [93.2- 96.0]	58.4% [52.3- 64.2]
Age						
15-24	20	52	75	11	87.2% [78.4- 92.9]	72.2% [60.9- 81.3]
25-34	48	68	314	27	92.1% [88.7- 94.5]	58.6% [49.5- 67.2]
35-49	41	33	567	15	97.4% [95.8- 98.5]	44.6% [33.8- 55.9]
Male	32	135	378	34	91.7% [88.7- 94.1]	80.8% [74.2- 86.1]
Age						
15-24	1	20	11	4	73.3% [47.6- 89.5]	95.2% [75.6-100.0]
25-34	15	64	90	14	86.5% [78.5- 91.9]	81.0% [70.9- 88.3]
35-49	16	51	277	16	94.5% [91.3- 96.7]	76.1% [64.6- 84.8]

Supplementary Table S9: Self-reported ART use and viral suppression in RCCS participants with HIV.

Contribution from male sources to incidence			Median age of male sources			Median age of female sources			Counterfactual additional number of men suppressed			Counterfactual reduction in incidence in female		
Round 10	Round 14	Round 18	Round 10	Round 14	Round 18	Round 10	Round 14	Round 18	Closing half the suppression gap	Closing the suppression gap	95-95-95 in men	Closing half the suppression gap	Closing the suppression gap	95-95-95 in men
**Central analysis**														
57.9%	61.4%	62.8%	28.5	30.1	33.5	25.0	26.8	26.0	75.1	150.2	172.6	25.1%	50.6%	58.4%
[56.2-59.6]	[59.8-63.1]	[60.2-65.2]	[22.8-40.2]	[22.6-41.0]	[23.6-41.6]	[18.0-36.2]	[19.7-37.2]	[19.0-36.4]	[53.9-96.4]	[107.8-192.8]	[136.8-210.3]	[24.2-26.2]	[48.6-52.8]	[54.9-61.7]
**Sensitivity analyses**														
Using incidence rates estimated with LOESS regression														
61.5%	57.5%	62.1%	27.7	31.8	34.0	24.0	25.0	26.0	75.1	150.2	172.6	25.3%	50.9%	58.1%
[59.5-63.5]	[55.5-59.5]	[60.4-63.9]	[22.3-38.8]	[23.1-42.8]	[23.8-41.6]	[18.0-34.8]	[18.9-36.0]	[18.3-35.9]	[53.9-96.4]	[107.8-192.8]	[136.8-210.3]	[24.1-26.6]	[48.5-53.6]	[53.6-62.1]
Using incidence rates estimated on a data subset to 28 continuously surveyed communities														
58.2%	62.3%	64.3%	29.5	31.0	34.0	25.0	27.7	27.0	75.1	150.2	172.6	25.5%	51.5%	56.4%
[56.5-59.8]	[60.6-64.0]	[61.6-66.9]	[23.0-41.1]	[23.0-42.1]	[24.2-43.0]	[18.0-36.4]	[19.4-37.2]	[19.0-37.0]	[53.9-96.4]	[107.8-192.8]	[136.8-210.3]	[24.3-26.9]	[48.9-54.3]	[52.0-60.6]
Using non-refined infection time estimates														
57.9%	61.4%	62.8%	28.1	30.0	33.4	24.5	26.0	25.8	75.1	150.2	172.6	25.1%	50.6%	58.3%
[56.1-59.6]	[59.8-63.0]	[60.3-65.2]	[22.6-40.2]	[22.3-41.3]	[23.7-42.0]	[18.0-36.3]	[19.6-37.4]	[19.0-36.5]	[53.9-96.4]	[107.8-192.8]	[136.8-210.3]	[24.0-26.3]	[48.3-53.0]	[54.6-62.0]
Without source-recipients pairs for which the source or recipient was sequenced after round 17														
58.0%	61.4%	62.8%	28.0	29.8	32.9	25.2	27.0	26.0	75.1	150.2	172.6	24.8%	50.0%	59.4%
[56.2-59.7]	[59.8-63.1]	[60.2-65.2]	[22.7-40.0]	[22.4-40.9]	[23.0-41.5]	[18.1-36.7]	[20.0-37.6]	[19.7-36.7]	[53.9-96.4]	[107.8-192.8]	[136.8-210.3]	[23.7-26.0]	[47.7-52.4]	[55.6-63.1]
Without source-recipients pairs for which the source or recipient was sequenced after round 16														
58.0%	61.4%	62.7%	28.0	30.0	33.0	24.7	26.0	25.0	75.1	150.2	172.6	24.9%	50.1%	59.0%
[56.3-59.7]	[59.8-63.0]	[60.2-65.2]	[22.8-39.9]	[22.6-40.8]	[23.9-41.1]	[18.0-36.5]	[19.8-37.6]	[19.0-36.2]	[53.9-96.4]	[107.8-192.8]	[136.8-210.3]	[23.6-26.2]	[47.5-52.8]	[55.0-63.1]
Without source-recipients pairs for which the source or recipient was sequenced after round 15														
58.0%	61.4%	62.8%	28.1	30.0	33.4	25.0	26.9	25.6	75.1	150.2	172.6	24.9%	50.2%	58.7%
[56.2-59.7]	[59.7-63.0]	[60.3-65.2]	[22.8-39.6]	[22.5-40.7]	[24.0-41.2]	[18.0-37.0]	[19.6-38.0]	[19.0-36.8]	[53.9-96.4]	[107.8-192.8]	[136.8-210.3]	[23.6-26.3]	[47.5-52.9]	[54.5-63.0]
Using a bootstrap sample of the source-recipient pairs (first draw)														
58.0%	61.4%	62.8%	29.2	31.0	34.0	25.4	27.1	29.0	75.1	150.2	172.6	25.4%	51.2%	57.0%
[56.2-59.7]	[59.8-63.0]	[60.2-65.2]	[23.0-40.3]	[23.0-41.1]	[24.1-42.0]	[18.8-36.1]	[19.8-37.0]	[20.0-36.0]	[53.9-96.4]	[107.8-192.8]	[136.8-210.3]	[24.5-26.4]	[49.2-53.2]	[53.9-60.2]
Using a bootstrap sample of the source-recipient pairs (second draw)														
57.8%	61.4%	62.7%	29.5	31.0	31.4	24.0	26.0	25.0	75.1	150.2	172.6	24.4%	49.1%	60.8%
[56.1-59.5]	[59.7-63.0]	[60.3-65.2]	[23.0-40.0]	[23.0-40.6]	[23.0-41.1]	[18.0-36.8]	[19.4-37.8]	[19.0-35.3]	[53.9-96.4]	[107.8-192.8]	[136.8-210.3]	[23.4-25.3]	[47.1-51.0]	[57.5-64.1]
Using a bootstrap sample of the source-recipient pairs (third draw)														
57.9%	61.4%	62.7%	29.0	31.0	34.0	24.6	26.4	25.0	75.1	150.2	172.6	25.5%	51.4%	57.3%
[56.1-59.5]	[59.7-63.1]	[60.3-65.2]	[22.7-40.5]	[22.4-41.3]	[23.0-42.0]	[18.0-36.9]	[19.4-37.9]	[19.0-36.7]	[53.9-96.4]	[107.8-192.8]	[136.8-210.3]	[24.4-26.7]	[49.2-53.8]	[53.4-61.0]

Supplementary Table S10: Sensitivity analyses.

LaTeX template

Contribution from male sources to incidence			Median age of male sources			Median age of female sources			Counterfactual additional number of men suppressed			Counterfactual reduction in incidence in female		
Round 10	Round 14	Round 18	Round 10	Round 14	Round 18	Round 10	Round 14	Round 18	Closing half the suppression gap	Closing the suppression gap	95-95-95 in men	Closing half the suppression gap	Closing the suppression gap	95-95-95 in men
Assuming an alternative form of the transmission rate (12a))														
60.3%	60.7%	64.3%	29.0	31.7	35.0	24.6	26.4	26.0	75.1	150.2	172.6	25.7%	52.0%	55.3%
[59.3-61.3]	[59.8-61.6]	[63.0-65.9]	[23.0-44.9]	[23.0-48.6]	[24.1-43.4]	[17.9-36.3]	[19.3-37.4]	[19.0-36.7]	[53.9-96.4]	[107.8-192.8]	[136.8-210.3]	[24.8-26.6]	[50.2-53.7]	[52.3-59.5]
Assuming an alternative form of the transmission rate (12b))														
60.5%	60.6%	63.8%	30.0	32.0	33.0	24.5	26.3	25.2	75.1	150.2	172.6	24.9%	50.3%	57.0%
[59.5-61.4]	[59.7-61.5]	[62.5-65.1]	[23.0-42.5]	[23.0-46.4]	[23.0-46.5]	[17.8-36.4]	[19.3-37.5]	[18.8-36.7]	[53.9-96.4]	[107.8-192.8]	[136.8-210.3]	[24.2-25.6]	[48.8-51.8]	[54.0-60.5]
Assuming an alternative form of the transmission rate (12c))														
60.4%	60.6%	64.0%	29.7	32.0	33.5	24.6	26.3	26.0	75.1	150.2	172.6	25.0%	50.6%	56.9%
[59.4-61.3]	[59.7-61.5]	[62.7-65.4]	[23.0-43.8]	[23.0-48.0]	[23.3-44.8]	[17.9-36.4]	[19.4-37.5]	[19.0-36.8]	[53.9-96.4]	[107.8-192.8]	[136.8-210.3]	[24.1-26.1]	[48.7-52.7]	[53.4-60.5]
Assuming an alternative form of the transmission rate (12d))														
57.9%	61.4%	62.7%	29.0	30.5	32.9	25.0	26.5	25.8	75.1	150.2	172.6	24.9%	50.2%	59.1%
[56.2-59.6]	[59.8-63.0]	[60.2-65.2]	[23.0-40.1]	[22.9-40.9]	[23.0-42.0]	[18.0-36.1]	[19.7-37.1]	[19.0-36.5]	[53.9-96.4]	[107.8-192.8]	[136.8-210.3]	[24.1-25.7]	[48.6-51.9]	[56.2-61.9]
Assuming an alternative form of the transmission rate (12e))														
58.0%	61.4%	62.8%	29.0	30.5	33.0	25.0	26.7	25.9	75.1	150.2	172.6	24.9%	50.2%	58.9%
[56.3-59.7]	[59.7-63.0]	[60.2-65.2]	[23.0-40.2]	[22.9-41.0]	[23.0-42.0]	[18.0-36.2]	[19.8-37.2]	[19.0-36.6]	[53.9-96.4]	[107.8-192.8]	[136.8-210.3]	[24.1-25.7]	[48.6-51.9]	[56.0-61.8]
Assuming an alternative form of the transmission rate (12f))														
57.9%	61.4%	62.8%	28.9	30.4	33.0	25.0	26.6	26.0	75.1	150.2	172.6	25.0%	50.3%	58.9%
[56.2-59.6]	[59.8-63.1]	[60.3-65.2]	[23.0-40.1]	[22.8-40.9]	[23.0-41.9]	[18.0-36.2]	[19.7-37.2]	[19.0-36.6]	[53.9-96.4]	[107.8-192.8]	[136.8-210.3]	[24.1-25.9]	[48.4-52.3]	[55.7-62.1]
Assuming the same proportion of viral suppression among non-participants as among participants of the same age, gender, and survey round														
57.9%	61.4%	62.8%	28.6	30.0	33.0	25.0	26.7	25.9	71.7	143.3	143.5	26.7%	53.6%	52.2%
[56.2-59.6]	[59.7-63.0]	[60.2-65.2]	[22.8-40.2]	[22.6-40.9]	[23.6-41.5]	[18.0-36.3]	[19.6-37.2]	[19.0-36.5]	[54.6-89.5]	[109.3-179.0]	[114.1-175.7]	[26.1-27.2]	[52.4-54.7]	[47.0-57.1]
Assuming that non-participants are not suppressed														
58.0%	61.4%	62.7%	28.5	30.1	34.0	25.0	26.8	26.3	254.7	329.9	351.9	52.3%	68.1%	74.6%
[56.3-59.7]	[59.8-63.0]	[60.2-65.2]	[22.8-40.1]	[22.5-41.1]	[24.0-42.0]	[18.0-36.2]	[19.8-37.2]	[19.0-37.9]	[232.7-275.5]	[300.0-358.6]	[333.4-372.5]	[50.0-54.6]	[65.8-70.5]	[73.4-75.8]
Assuming that prevalence in non-participants is 25% higher than in participants														
58.0%	61.5%	62.7%	28.6	30.1	33.5	25.0	26.8	26.0	81.9	163.9	189.3	25.2%	50.7%	58.3%
[56.3-59.7]	[59.8-63.1]	[60.2-65.1]	[22.8-40.2]	[22.6-41.0]	[23.7-41.6]	[18.0-36.3]	[19.7-37.2]	[19.0-36.4]	[58.9-105.1]	[117.7-210.1]	[150.4-230.4]	[24.2-26.2]	[48.6-52.9]	[54.8-61.7]
Assuming that prevalence in men non-participants is 25% higher than in men participants														
58.2%	61.6%	62.9%	28.6	30.1	33.5	25.0	26.7	26.0	81.9	163.9	189.3	25.1%	50.6%	58.4%
[56.5-59.8]	[60.0-63.3]	[60.4-65.4]	[22.8-40.2]	[22.6-41.0]	[23.6-41.6]	[18.0-36.2]	[19.6-37.2]	[19.0-36.4]	[58.9-105.1]	[117.7-210.1]	[150.4-230.4]	[24.1-26.2]	[48.5-52.9]	[54.9-61.8]
Assuming that prevalence in women non-participants is 25% higher than in women participants														
57.8%	61.2%	62.5%	28.5	30.0	33.4	25.0	26.8	26.0	75.1	150.2	172.6	25.1%	50.6%	58.4%
[56.0-59.4]	[59.6-62.8]	[60.0-64.9]	[22.8-40.2]	[22.6-41.0]	[23.6-41.6]	[18.0-36.3]	[19.7-37.2]	[19.0-36.4]	[53.9-96.4]	[107.8-192.8]	[136.8-210.3]	[24.1-26.2]	[48.5-52.8]	[55.0-61.7]
Defining viral suppression as a viral load measurement below 200 copies/mL plasma blood														
57.9%	61.4%	62.8%	28.6	30.2	33.1	25.0	27.0	26.0	73.2	146.4	197.2	22.8%	46.0%	61.6%
[56.2-59.6]	[59.7-63.0]	[60.3-65.2]	[22.9-40.2]	[22.6-41.0]	[23.6-41.5]	[18.0-36.3]	[19.7-37.3]	[19.1-36.6]	[51.7-94.5]	[103.5-189.1]	[161.7-234.6]	[21.7-24.0]	[43.7-48.5]	[58.1-64.9]
Without adjustments for potentially unequal sampling of sources														
57.9%	61.4%	62.8%	29.0	30.6	33.7	25.0	26.3	26.0	75.1	150.2	172.6	25.2%	50.6%	58.4%
[56.1-59.6]	[59.8-63.1]	[60.2-65.2]	[22.7-40.0]	[22.5-40.8]	[23.5-41.4]	[18.0-36.1]	[19.7-37.2]	[19.0-36.2]	[53.9-96.4]	[107.8-192.8]	[136.8-210.3]	[24.2-26.2]	[48.7-52.7]	[55.1-61.6]

Supplementary Table S10: **Sensitivity analyses (continued).**

References

[1] Gall, A. *et al.* Universal amplification, next-generation sequencing, and assembly of HIV-1 genomes. *Journal of Clinical Microbiology* **50**, 3838–3844 (2012) .

[2] Bonsall, D. *et al.* A comprehensive genomics solution for hiv surveillance and clinical monitoring in low-income settings. *Journal of Clinical Microbiology* **58**, e00382–20 (2020) .
