# Supplementary material for: Longitudinal population-level HIV epidemiologic and genomic surveillance highlights growing gender disparity of HIV transmission in Uganda
Source: Nat Microbiol. 2023 Dec 5;9(1):35–54. doi: 10.1038/s41564-023-01530-8 (PMC10769880; doi:10.1038/s41564-023-01530-8)
Supplement: Supplementary file 2 — Reporting Summary [file 41564_2023_1530_MOESM2_ESM.pdf]

nature portfolio

Corresponding author(s): Oliver Ratmann

Last updated by author(s): Nov 13, 2023

Reporting Summary

Nature Portfolio wishes to improve the reproducibility of the work that we publish. This form provides structure for consistency and transparency in reporting. For further information on Nature Portfolio policies, see our Editorial Policies and the Editorial Policy Checklist.

Statistics

For all statistical analyses, confirm that the following items are present in the figure legend, table legend, main text, or Methods section.

n/a	Confirmed	
☐	☒	The exact sample size (*n*) for each experimental group/condition, given as a discrete number and unit of measurement
☐	☒	A statement on whether measurements were taken from distinct samples or whether the same sample was measured repeatedly
☐	☒	The statistical test(s) used AND whether they are one- or two-sided<br>*Only common tests should be described solely by name; describe more complex techniques in the Methods section.*
☐	☒	A description of all covariates tested
☐	☒	A description of any assumptions or corrections, such as tests of normality and adjustment for multiple comparisons
☐	☒	A full description of the statistical parameters including central tendency (e.g. means) or other basic estimates (e.g. regression coefficient) AND variation (e.g. standard deviation) or associated estimates of uncertainty (e.g. confidence intervals)
☐	☒	For null hypothesis testing, the test statistic (e.g. *F*, *t*, *r*) with confidence intervals, effect sizes, degrees of freedom and *P* value noted<br>*Give P values as exact values whenever suitable.*
☐	☒	For Bayesian analysis, information on the choice of priors and Markov chain Monte Carlo settings
☐	☒	For hierarchical and complex designs, identification of the appropriate level for tests and full reporting of outcomes
☒	☐	Estimates of effect sizes (e.g. Cohen's *d*, Pearson's *r*), indicating how they were calculated

Our web collection on statistics for biologists contains articles on many of the points above.

Software and code

Policy information about availability of computer code

Data collection	For sequencing, the following software were used, QuantStudio Real-Time PCR System v1.3, Agilent TapeStation Software Analysis 4.1.1, Clarity Version 4.2.23.287, FreezerPro® (7.4.0-r14598), LabArchives ELN (Electronic Lab Notebook) 2023. All epidemiologic data collected through the Rakai Community Cohort Study are stored in a database running Microsoft SQL server 2019 and Microsoft Access version 2016.
Data analysis	All data were analyzed with R version 4.1.2, the R package stats version 3.6.2, the R package Rstan version 2.21.0, the R package cmdstanR version 0.5.1, the R package mgcv version 1.8-38, shiver version 1.5.7, phyloscanner version 1.8.1, MAFFT version 7.475, IQ-Tree version 2.0.3, phyloTSI version 1.0.0, ; and using the custom code scripts freely available at https://github.com/MLGlobalHealth/phyloSI-RakaiAgeGender. Team communications were supported through the Zulip chat app 5.10.2 (https://zulip.com/).

For manuscripts utilizing custom algorithms or software that are central to the research but not yet described in published literature, software must be made available to editors and reviewers. We strongly encourage code deposition in a community repository (e.g. GitHub). See the Nature Portfolio guidelines for submitting code & software for further information.

Data

Policy information about availability of data

All manuscripts must include a data availability statement. This statement should provide the following information, where applicable:

- Accession codes, unique identifiers, or web links for publicly available datasets
- A description of any restrictions on data availability
- For clinical datasets or third party data, please ensure that the statement adheres to our policy

Pseudo-anonymised data from the RCCS incidence and transmission cohort as well as pseudo-anonymised deep-sequence phylogenies to reproduce all analyses are available from Zenodo (https:/zenodo.org/record/8412741) as open-access data set under the CC-BY-4.0 license. HIV consensus sequences are available from Zenodo (https://zenodo.org/records/10075815) and the PANGEA-HIV sequence repository (https://github.com/PANGEA-HIV/PANGEA-Sequences) as open-access data set under the CC-BY-4.0 license, with identifiers changed to ensure participants cannot be identified from this data set.

Additional deep-sequence HIV-1 reads can be requested from PANGEA-HIV under a managed access policy due to privacy and ethical reasons, as the risks to the participants outweigh the benefits. The risk is to accidentally disclose evidence of transmission, or of not transmission, therefore making light evidence of sexual contact and transmission that could jeopardise relationships, and in some instances lead to criminalisation which is against UNAIDS ethical guidelines. The process for accessing data, the PANGEA-HIV Data Sharing Policy and a detailed description of what data are available is laid out in full at (https://www.pangea-hiv.org/join-us). Briefly, applicants can apply to receive additional data by submitting a concept sheet proposal in which they explain the research question and how they will mitigate potential risks to participant privacy. In line with requirements for PANGEA members, applicants will be asked to present proof of human subject research training and comply with PANGEA-internal publication agreements. PANGEA encourages external applicants to collaborate with the researchers who generated the data. For more information contact PANGEA project manager Lucie Abeler-Dörner (lucie.abeler-dorner@bdi.ox.ac.uk). The time frame for a response to requests is 2-4 weeks.

Additional cohort data can be requested from RHSP. Because HIV transmission is criminalized in Uganda and due to further privacy considerations, RHSP maintains a controlled access data policy for corresponding epidemiological metadata and corresponding data collection tools. In brief, RHSP policy requires individuals to submit an RSHSP data request form (available upon request) and a brief concept note (1-2 pages) detailing their research questions and methods. In addition, researchers are asked to provide a curriculum vitae/resume along with proof of human subjects research training. Concept sheets can be submitted to Dr. Godfrey Kigozi (gkigozi@rhsp.org), executive director of the RHSP. Only individuals named on the original data request and who provide the request, CV/resume and HSR training, are permitted access to the data. Released data are not to be reused for other purposes outside of approved concepts. The time frame for a response to requests is 2-4 weeks.

Human research participants

Policy information about studies involving human research participants and Sex and Gender in Research.

Reporting on sex and gender	The results presented in this study derive from data collected through nine consecutive survey rounds of the Rakai Community Cohort Study (RCCS) between September 2003 and May 2018. Participants self-reported their gender, birth date, and age at visit.
Population characteristics	Following consent, participants reported on demographics, behavior, health, and health service use. All participants were offered free voluntary counseling and HIV testing as part of the survey. Rapid tests at the time of the survey and confirmatory enzyme immunoassays were performed to determine HIV status. All participants were provided with pre-test and post-test counseling, and referrals of individuals who were HIV-positive for ART. Additionally, all consenting participants, irrespective of HIV status, were offered a venous blood sample for storage/future testing, including viral phylogenetic studies. Table S1 summarises the characteristics of the RCCS participants and HIV-positive participants by age and gender. Within the RCCS, we also performed population-based HIV deep-sequencing spanning a period of more than 6 years, from August 2011 to April 2018. The primary purpose of viral deep sequencing was to reconstruct transmission networks and identify the population-level sources of infections, thus complementing the data collected through the incidence cohort. Participants are characterised in Supplementary Table S1.
Recruitment	For each survey round, the RCCS did a household census, and subsequently invited all individuals that were of age 15-49 years and residents for at least 1 month to participate in the open, longitudinal RCCS survey. Eligible individuals first attended group consent procedures, and individual consent was obtained privately by a trained RCCS interviewer. While our analyses accounted for participation biases by age, gender and community we cannot rule out the possibility that other unmeasured factors associated with age and gender and also HIV serostatus, viral load suppression, and onward transmission may have been related to study participation, potentially biasing results.
Ethics oversight	The study was independently reviewed and approved by the Ugandan Virus Research Institute, Scientific Research and Ethics Committee, protocol GC/127/13/01/16; the Ugandan National Council of Science and Technology; and the Western Institutional Review Board, protocol 200313317. All study participants provided written informed consent at baseline and follow-up visits using institutional review board approved forms. This project was reviewed in accordance with CDC human research protection procedures and was determined to be research, but CDC investigators did not interact with human subjects or have access to identifiable data or specimens for research purposes. Participants in the RCCS received 10,000UGX (approximately 2.50USD) in compensation for the baseline and follow-up surveys.

Note that full information on the approval of the study protocol must also be provided in the manuscript.

Field-specific reporting

Please select the one below that is the best fit for your research. If you are not sure, read the appropriate sections before making your selection.

☐ Life sciences ☒ Behavioural & social sciences ☐ Ecological, evolutionary & environmental sciences

For a reference copy of the document with all sections, see nature.com/documents/nr-reporting-summary-flat.pdf

Behavioural & social sciences study design

All studies must disclose on these points even when the disclosure is negative.

Study description	The results presented in this study derive from data collected through nine consecutive survey rounds of the Rakai Community Cohort Study (RCCS) between September 2003 and May 2018. All data collected through the surveys were of quantitative nature.
Research sample	Individuals that were of age 15-49 years and residents for at least 1 month in inland and fishing communities in South-central Uganda. In total, 38749 participants were enrolled. Participants are characterized by survey round, gender and age in Supplementary Table S1. Sampling was representative of the population except for individuals away for work or at school.
Sampling strategy	The entire eligible population was invited to participate in the RCCS; sampling was thus population-based and survey participation was voluntary. Viral sequencing was performed on plasma samples from participants with HIV who had no viral load measurement and self-reported being ART-naïve at the time of the survey, or who had a viral load measurement above 1,000 copies/mL plasma.
Data collection	Between September 2003 and May 2018, nine consecutive survey rounds of the Rakai Community Cohort Study (RCCS) were conducted in 36 inland communities in south-central Uganda. For each survey round, the RCCS did a household census, and subsequently invited all individuals that were of age 15-49 years and residents for at least 1 month to participate in the open, longitudinal RCCS survey; and so data collection was not randomized, and data collection was blind relative to previous interactions with individuals or any personal characteristics apart from age and residency status, and any research questions. Eligible individuals first attended group consent procedures, and individual consent was obtained privately by a trained RCCS interviewer. Following consent, participants reported in a private location, typically a tent at the survey hub, on demographics, behavior, health, and health service use. All participants were offered free voluntary counseling and HIV testing as part of the survey. Rapid tests at the time of the survey and confirmatory enzyme immunoassays were performed to determine HIV status. All participants were provided with pre-test and post-test counseling, and referrals of individuals who were HIV-positive for ART. Additionally, all consenting participants, irrespective of HIV status, were offered a venous blood sample for storage/future testing, including viral phylogenetic studies. All epidemiologic data collected through RCCS are stored in a database running Microsoft SQL server 2019 and Microsoft Access version 2016. Further details for the survey methods are described in Grabowski, M. K. et al. HIV prevention efforts and incidence of HIV in Uganda. New England Journal of Medicine 377, 2154–2166 (2017)
Timing	Surveys were conducted between September 2003 and May 2018. The first survey round considered in this analysis was Round 10, September 26, 2003 - November 23, 2004; followed by Round 11, February 15, 2005 - June 30, 2006; Round 12, August 30, 2006 - June 06, 2008; Round 13, June 17, 2008 - July 12, 2009; Round 14, January 18, 2010 - June 21, 2011; Round 15, August 10, 2011 - July 05, 2013; Round 16, July 08, 2013 - January 30, 2015; Round 17, February 23, 2015 - September 02, 2016; and Round 18, October 03, 2016 - May 22, 2018.
Data exclusions	Viral sequence data were excluded if they had not sufficient depth or length for the purpose of deep-sequence phylogenetic analyses. We required that individuals had a depth of ≥ 30 such reads over at least 3 non-overlapping 250bp genomic windows. 88 transmission pairs had to be excluded due to ethical considerations.
Non-participation	Non-participation rates among eligible individuals in the communities considered were as follows. Round 10: 4,569/11,976; Round 11 4,255/12,528; Round 12 4,966/13,718; Round 13 4,715/13,433; Round 14 5,165/14,828; Round 15 7,217/20,806; Round 16 7,815/21,887; Round 17 7,836/22,929; Round 18 8,216/23,269. Participation rates varied by age and gender and are described in Supplementary Table S1 and Extended Data Fig 1, and the most common reason for non-participation was being away for work or school.
Randomization	Participants were not allocated into experimental groups.

Reporting for specific materials, systems and methods

We require information from authors about some types of materials, experimental systems and methods used in many studies. Here, indicate whether each material, system or method listed is relevant to your study. If you are not sure if a list item applies to your research, read the appropriate section before selecting a response.

Materials & experimental systems

n/a	Involved in the study
☒	☐ Antibodies
☒	☐ Eukaryotic cell lines
☒	☐ Palaeontology and archaeology
☒	☐ Animals and other organisms
☒	☐ Clinical data
☒	☐ Dual use research of concern

Methods

n/a	Involved in the study
☒	☐ ChIP-seq
☒	☐ Flow cytometry
☒	☐ MRI-based neuroimaging
